# Zebrafish *slc30a10* deficiency revealed a novel compensatory mechanism of Atp2c1 in maintaining manganese homeostasis

**DOI:** 10.1371/journal.pgen.1006892

**Published:** 2017-07-10

**Authors:** Zhidan Xia, Jiayu Wei, Yingniang Li, Jia Wang, Wenwen Li, Kai Wang, Xiaoli Hong, Lu Zhao, Caiyong Chen, Junxia Min, Fudi Wang

**Affiliations:** 1 Nutrition Discovery Innovation Center, Institute of Nutrition and Food Safety, School of Public Health, The First Affiliated Hospital, Institute of Translational Medicine, The Children's Hospital, Collaborative Innovation Center for Diagnosis and Treatment of Infectious Diseases, School of Medicine, Zhejiang University, Hangzhou, China; 2 Department of Nutrition, Precision Nutrition Innovation Center, School of Public Health, Zhengzhou University, Zhengzhou, China; 3 College of Life Science, Zhejiang University, Hangzhou, China; Marine Biological Laboratory, UNITED STATES

## Abstract

Recent studies found that mutations in the human *SLC30A10* gene, which encodes a manganese (Mn) efflux transporter, are associated with hypermanganesemia with dystonia, polycythemia, and cirrhosis (HMDPC). However, the relationship between Mn metabolism and HMDPC is poorly understood, and no specific treatments are available for this disorder. Here, we generated two zebrafish *slc30a10* mutant lines using the CRISPR/Cas9 system. Compared to wild-type animals, mutant adult animals developed significantly higher systemic Mn levels, and Mn accumulated in the brain and liver of mutant embryos in response to exogenous Mn. Interestingly, *slc30a10* mutants developed neurological deficits in adulthood, as well as environmental Mn-induced manganism in the embryonic stage; moreover, mutant animals had impaired dopaminergic and GABAergic signaling. Finally, mutant animals developed steatosis, liver fibrosis, and polycythemia accompanied by increased *epo* expression. This phenotype was rescued partially by EDTA- CaNa_2_ chelation therapy and iron supplementation. Interestingly, prior to the onset of *slc30a10* expression, expressing ATP2C1 (ATPase secretory pathway Ca^2+^ transporting 1) protected mutant embryos from Mn exposure, suggesting a compensatory role for Atp2c1 in the absence of Slc30a10. Notably, expressing either wild-type or mutant forms of SLC30A10 was sufficient to inhibit the effect of ATP2C1 in response to Mn challenge in both zebrafish embryos and HeLa cells. These findings suggest that either activating ATP2C1 or restoring the Mn-induced trafficking of ATP2C1 can reduce Mn accumulation, providing a possible target for treating HMDPC.

## Author summary

Impaired function of the manganese transporter SLC30A10 has been implicated in HMDPC (hypermanganesemia with dystonia, polycythemia, and cirrhosis), an early-onset metabolic disorder clinically characterized by increased systemic Mn levels, neurological impairment, polycythemia, and hepatic injury. No specific treatment is currently available for HMDPC. Moreover, the mechanisms that underlie Mn metabolism are poorly understood, thereby hindering the development of effective treatments. To investigate the physiological processes underlying Mn metabolism and to develop new disease models of HMDPC, we generated two zebrafish *slc30a10* mutant lines using the CRISPR/Cas9 system and found that these mutants develop clinical deficits typically associated with HMDPC. Furthermore, we identified a putative compensatory role for ATP2C1 in the absence of SLC30A10 with respect to modulating Mn metabolism. These findings provide a valuable tool for investigating the role of manganese dysregulation in neurological degenerative diseases and which can be used to develop new pharmacological approaches for managing Mn accumulation.

## Introduction

Manganese (Mn) is an essential element required for the catalytic activity of numerous cellular enzymes [1]. The mammalian divalent metal ion influx transporter SLC39A8 has high affinity for Mn [2,3], and mutations are associated with low blood Mn levels and impaired function of Mn-dependent enzymes, including β-1,4-galactosyltransferase, thus leading to congenital glycosylation defects and impaired growth in infants [4,5]. On the other hand, high levels of Mn are toxic due to increased cellular oxidative stress, reduced mitochondrial function, and cell death [6]. In humans, the major source of Mn is dietary Mn absorbed through the intestinal wall; excess Mn is removed from the body via the hepatobiliary route [7,8]. Under chronic conditions of high Mn, Mn either overloads the liver (causing hepatic dysfunction) or increases systemic toxicity, inducing an irreversible and incurable parkinsonian-like syndrome in which the vulnerable basal ganglia are affected by accumulated Mn [8–10].

This Mn-induced parkinsonian-like syndrome can occur due to increased environmental exposure or in certain occupational settings [11,12]. In addition, high Mn levels due to parenteral nutrition and/or drug addiction can also lead to disease [13,14]. Furthermore, patients who have impaired liver function and are unable to adequately excrete Mn are sensitive to high Mn and can develop Mn-induced parkinsonian-like symptoms [15,16]. For example, reduced function of the transporter SLC39A14, which mediates the hepatic uptake of Mn for subsequent biliary excretion, causes cerebral Mn accumulation due to increased systemic Mn levels [17].

Several transporters play an important role in Mn metabolism, including importers such as transferrin, SLC39A8, and SLC39A14, and exporters such as ATP13A2, ATP2C1, and SLC30A10 [1]. However, how these proteins coordinate in order to maintain Mn homeostasis is poorly understood.

Hypermanganesemia with dystonia, polycythemia, and cirrhosis (HMDPC, OMIM# 613280) was the first Mn metabolism disorder to be linked to a genetic defect [18–20]. Specifically, autosomal recessive mutations in the *SLC30A10* gene, which encodes a key Mn exporter [21], have been linked to HMDPC [22–24]. To date, 28 affected individuals in 14 families have been described [25,26]. The majority of these 28 patients presented with dystonia (25 patients) or spastic paraparesis (one patient); the remaining two patients developed late-onset parkinsonism [26]. However, the mechanism by which SLC30A10 contributes to Mn homeostasis, and the underlying pathological processes associated with *SLC30A10* mutations, are unknown.

Currently, the preferred treatment for HMDPC is chelation therapy with intravenous disodium calcium edetate (EDTA-CaNa_2_), a treatment commonly used for environmental Mn intoxication [22,27]. Although chelation therapy improves the neurological symptoms, reduces serum Mn accumulation, and prevents further hepatic pathology [18,28], it has the disadvantage of requiring strict monitoring of other essential minerals, including zinc, copper, and selenium [22]. Moreover, intravenous EDTA-CaNa_2_ and close patient monitoring are generally not available in most regions with inadequate medical resources [26]. Chelation therapy also requires lifelong treatments of at least two 5-day courses per month, and once treatment has begun, reducing and/or stopping treatment can cause the patient’s condition to worsen [29]. In addition to chelation therapy, oral iron supplementation can reduce intestinal Mn absorption by competing for common transporter proteins; however, the patient’s iron levels must be monitored closely in order to prevent iron toxicity [29,30]. Therefore, safer, more efficacious treatments and/or specific therapeutic strategies are urgently needed for patients with HMDPC and other Mn-associated conditions.

Here, we generated two *slc30a10* mutant zebrafish models using the CRISPR/Cas9 system. We found that mutant animals develop a phenotype strikingly similar to the typical clinical symptoms in patients with HMDPC. EDTA-CaNa_2_ chelation therapy and treatment with ferrous fumarate partially rescued the phenotype, suggesting that these models might serve as a valuable tool for screening new pharmacological approaches. Importantly, we also found that SLC30A10 inhibits the Mn-clearing function of ATP2C1, revealing a novel mechanism with respect to Mn metabolism and creating new opportunities for treating HMDPC and related conditions.

## Results

### Slc30a10 is a manganese transporter in zebrafish

As a first step toward determining whether zebrafish are a suitable model for studying Mn metabolism, we examined whether Mn is metabolized in the same organs in zebrafish as in humans. Wild-type zebrafish embryos were exposed to various concentrations of MnCl_2_ for 24 hours at different developmental stages, and their behavior was monitored the following day. We found that embryos are most sensitive to Mn exposure at 5–6 dpf (days post-fertilization); concentrations lower than 0.3 mM had no effect on swimming ability, whereas concentrations higher than 1 mM caused complete immobility in 100% of embryos (S1A Fig). Based on these preliminary data, we consider Mn concentrations lower than 0.3 mM and higher than 1 mM to be relatively low and relatively high, respectively. Importantly, embryos exposed to 1 mM MnCl_2_ developed dark-colored brain and liver tissues (S1B and S1C Fig), suggesting that these two organs are sensitive to Mn exposure in zebrafish just like humans [22,23,26].

Next, we measured Mn accumulation in the head, middle trunk, and tail of Mn-exposed embryos. We found higher levels of Mn accumulated in the head and middle trunk, and lower levels in the tail (S1D Fig). After transferring Mn-exposed embryos to fresh Mn-free Holt buffer, the color of both the liver and brain returned to baseline (S1E Fig), and Mn levels were reduced in these embryos (S1F Fig), suggesting that the accumulation of Mn in the brain and liver of wild-type embryos is reversible.

**Fig 1:**
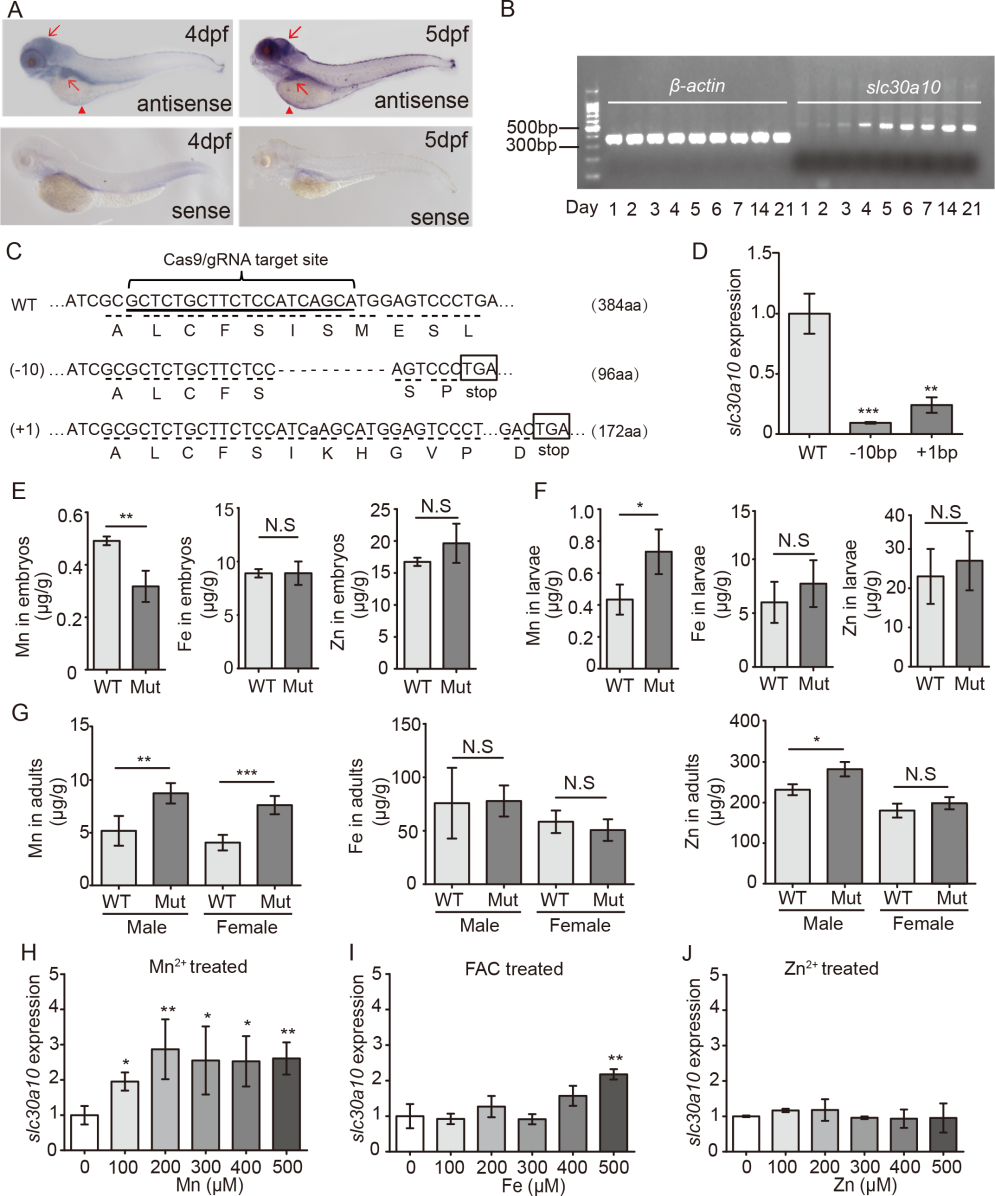
Slc30a10 functions as a Mn exporter in zebrafish. (A) *In situ* hybridization of wild-type zebrafish using the antisense *slc30a10* probe, showing expression in the brain and liver (red arrows), as well as the YSL (red arrowheads). A sense probe was used as a negative control. (B) Semi-quantitative RT-PCR of *slc30a10* mRNA in wild-type 1–7 dpf embryos, 14 dpf larvae, and 21 dpf larvae, showing the onset of expression at 5 dpf. (C) DNA and corresponding amino acid sequences of the wild-type (WT), 10-bp deletion (-10), and 1-bp insertion (+1) *slc30a10* alleles following CRISPR/Cas9-based editing. (D) Summary of *slc30a10* expression in WT and both *slc30a10* mutant lines (n = 3 sets of 50 embryos/group). (E-G) Mn, Fe, and Zn concentration was measured in wild-type and mutant 1-week-old embryos (E; n = 6 sets of 1000 embryos/group), 3-week-old larvae (F; n = 3 sets of 50 larvae/group), and 4-month-old adults (G; n = 3 adults/group). (H-J) Heterozygous embryos were exposed to the indicated concentrations of Mn^2+^ (H), Fe^3+^ (ferric ammonium citrate, FAC; I), or Zn^2+^ (J) for 24 hours at 5 dpf. At 6 dpf, *slc30a10* expression was measured; n = 3 sets of embryos/group. *p<0.05, **p<0.01, and ***p<0.001; N.S., not significant (*p*>0.05).

Next, we examined whether zebrafish models are suitable for studying HMDPC, a genetic condition associated with impaired function of the Mn transporter SLC30A10. By searching the zebrafish genomic database, we identified the zebrafish *slc30a10* gene as an ortholog of the human *SLC30A10* gene, with 42% identity and 55% similarity at the protein level (S2 Fig). Because *SLC30A10* is highly expressed in the human brain and liver, where it functions as a Mn exporter, we examined the expression pattern of *slc30a10* in zebrafish. We found that *slc30a10* is expressed in the brain, liver, and yolk syncytial layer (YSL) of zebrafish embryos (Fig 1A), with robust expression beginning at approximately 5 dpf (Fig 1B). These similarities between humans and zebrafish with respect to their Mn-sensitive organs and *SLC30A10* expression patterns (i.e., in the brain and liver) indicate that *slc30a10* mutant zebrafish might serve as a suitable model for HMDPC.

To create such a model, we generated two separate *slc30a10* knockout zebrafish lines using the CRISPR/Cas9 system. One line contains a 10-bp frameshift deletion at codon 95, which introduces a premature stop at codon 97. The other line contains a 1-bp frameshift insertion at codon 96, which introduces a premature stop at codon 173 (Fig 1C). Both mutant lines have significantly reduced *slc30a10* expression (Fig 1D; S1G Fig), and the residual transcripts correspond to the predicted mutant forms, which were confirmed by sequencing (S1H Fig). Because these two lines develop the same phenotype, all subsequent experiments were performed using the line carrying the 10-bp deletion.

To confirm that the zebrafish *slc30a10* gene encodes a Mn exporter, we measured the levels of several minerals in mutant animals. Severe hypermanganesemia—the primary metabolic abnormality in HMDPC—is associated with subtle perturbations in iron metabolism in some patients [22,23]. Moreover, the ZnT (SLC30) family of proteins primarily facilitates the efflux of zinc [31–33]. Therefore, we measured Mn, Fe, and Zn levels in embryonic, larval, and adult zebrafish using ICP-MS. Surprisingly, Mn levels were lower in 1-week-old mutant embryos compared to wild-type embryos; in contrast, both Fe and Zn levels were similar between wild-type and mutant embryos (Fig 1E). Because embryos are nourished exclusively by the yolk in this stage of development, the levels of trace minerals in these embryos reflect their absorption from the yolk. In 3-week-old larvae, on the other hand, we found higher Mn levels in the mutants compared to wild-type animals; as in the embryonic stage, both Fe and Zn were similar between groups (Fig 1F). These data indicate that Mn accumulates rapidly in mutant animals during the feeding stage. Finally, in 4-month-old adults, systemic Mn levels were significantly higher in both male and female mutant animals compared to wild-type animals, and Fe levels were similar between wild-type and mutant animals. Interestingly, Zn levels were higher in mutant animals compared to wild-type animals, but only in male animals (Fig 1G). We measured the levels of several other metals in 4-month-old adults, but found no additional differences between mutants and wild-type animals (S1 Table). Taken together, these results indicate that Slc30a10 functions primarily as a Mn exporter during zebrafish development.

### *Slc30a10* expression is regulated by Mn in zebrafish

We measured *slc30a10* expression in wild-type embryos exposed to various concentrations of MnCl_2_, ferric ammonium citrate (FAC), and ZnCl_2_. Even at a relatively high concentration (1 mM), none of the minerals tested caused a change in *slc30a10* expression.

Next, we measured the effect of mineral exposure on *slc30a10* expression in heterozygous embryos. Unlike wild-type animals, the heterozygous developed defects when exposed to Mn^2+^ or Fe^3+^ at concentrations higher than 500 μM and Zn^2+^ at concentrations higher than 250 μM.; therefore, we treated embryos with Mn^2+^, Fe^3+^, and Zn^2+^ concentrations below these levels. We found that *slc30a10* expression increased in heterozygous with increasing concentrations of Mn^2+^ (Fig 1H) and was increased only with the highest concentration of Fe^3+^ (Fig 1I); in contrast, *slc30a10* expression was not affected by Zn^2+^ at any concentration tested (Fig 1J). These data indicate that *slc30a10* expression is regulated primarily by Mn^2+^ in heterozygous embryos.

We also found that *slc30a10* expression increased in mutant adults following Mn exposure (S3A Fig). In addition, the expression of *slc30a10* in mutant adults was higher than in embryos; in contrast, *slc30a10* expression was unchanged between the embryonic stage and adulthood in wild-type animals (S3B Fig), suggests that the chronic accumulation of dietary Mn upregulates *slc30a10* expression in adult *slc30a10* mutants.

### *Slc30a10* mutant zebrafish develop neurological defects

**Fig 2:**
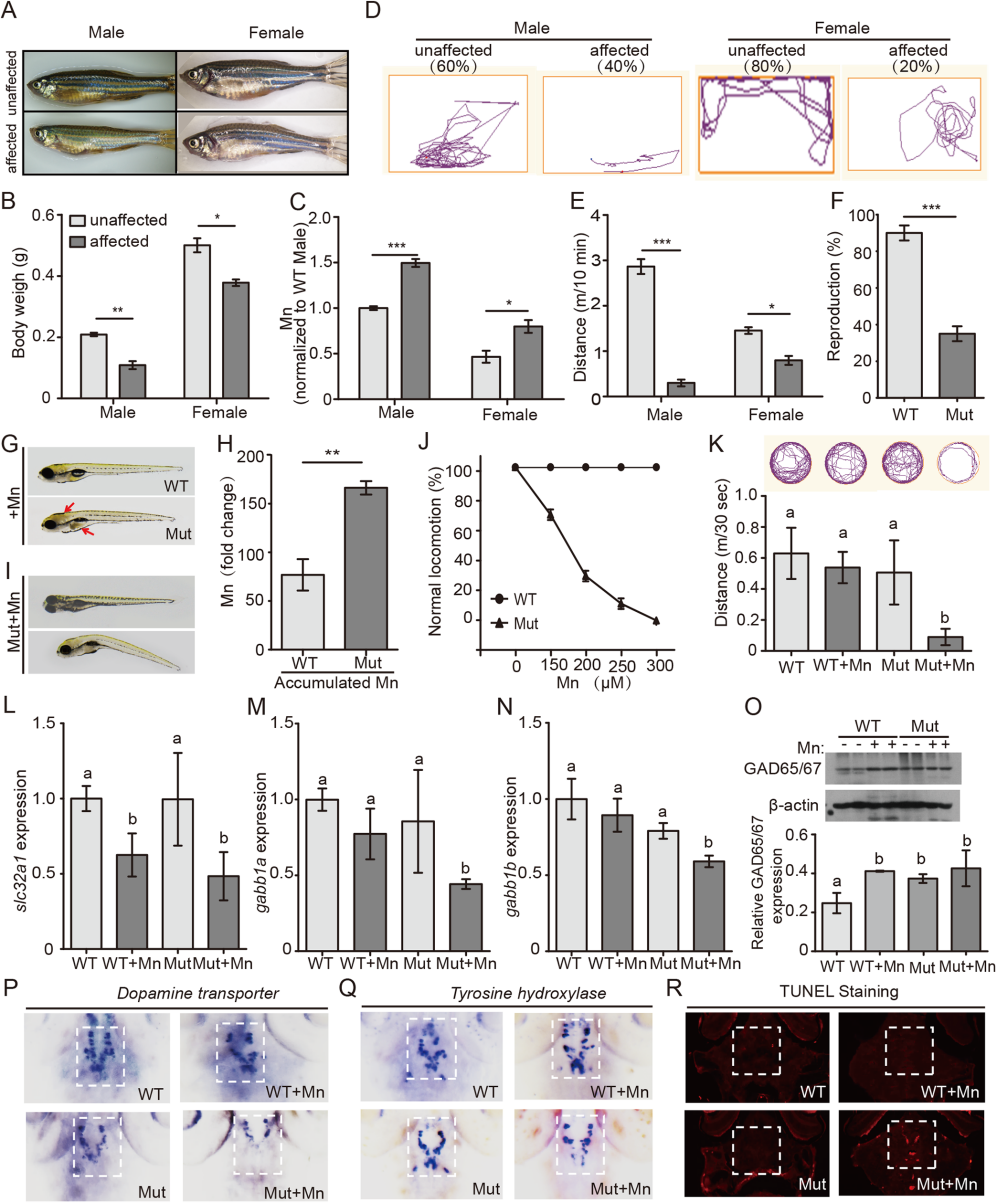
*Slc30a10* mutants develop impaired neurological function. (A) Example images of affected mutant adults and their unaffected siblings; note the thinner body shape of the affected siblings. (B) Summary of body weight of affected adults and unaffected siblings (n = 4 adults/group). (C) Affected mutants have higher Mn levels compared to their unaffected siblings (n = 4 adults/group). (D) Example movement traces of affected adults and their unaffected siblings. (E) Affected adults swim shorter distances than their unaffected siblings (n = 6 adults/group). (F) Summary of the reproductive capacity of WT and mutant adults (n = 20 adults/group). (G) Example images of a wild-type and mutant embryo, showing a darker color in the brain and liver (red arrows) in the mutant. (H) Mn exposure causes a larger increase in Mn accumulation in mutant animals compared to wild-type animals (n = 3 sets of 200 embryos/group). (I) Example images of two mutant embryos after Mn exposure. Note the distorted body shape of the Mn-treated mutant. (J) Locomotion (measured as swimming and a normal escape response) is reduced in mutant embryos following Mn treatment, whereas wild-type animals are not affected (n = 3 sets of 20 embryos/group). (K) Mutant embryos exposed to Mn swim a shorter distance than untreated mutants and wild-type embryos (n = 6 embryos/group). (L) *Slc32a1* expression is reduced in both wild-type and *slc30a10* embryos following Mn exposure (n = 3 sets of 20 embryos/group). (M-N) *Gabb1a* (M) and *gabb1b* (N) expression is reduced in Mn-exposed *slc30a10* mutants (n = 3 sets of 20 embryos/group). (O) Western blot analysis of Gad65/67 in wild-type and mutant embryos treated with or without Mn. (P-Q) *In situ* hybridization of *dat* and *th* in mutant embryos following Mn exposure, showing reduced expression of both genes compared to untreated mutants and WT embryos. (R) TUNEL staining in the brain of mutant embryos following Mn exposure, showing increased apoptosis compared to untreated mutants and WT embryos. *p<0.05, **p<0.01, and ***p<0.001; in K‒O, groups with different letters differed significantly (*p*<0.05).

Neurological disorders, including impaired gait and speech, are the most common clinical features among patients with HMDPC. Similarly, our *slc30a10* mutant zebrafish begin to develop neurological defects at approximately 4 months of age. Within the first year, approximately 40% and 20% of the mutant males and females, respectively, are affected; by the second year, these percentages increase to approximately 70% and 40%, respectively. In general, the affected mutant animals are smaller and thinner than their unaffected siblings (Fig 2A and 2B). In addition, our ICP-MS analysis revealed that the affected mutants have higher levels of Mn compared to unaffected siblings (Fig 2C).

In addition to morphological changes, affected mutants have a distinct bradykinesia-like swimming pattern (S1 and S2 Movies). Thus, the affected mutants move slowly, less frequently, and tend to stay at the bottom of the tank. To better characterize the swimming pattern of these affected mutants, we used behavior-tracking software, which confirmed that the affected mutants stayed in a relatively limited area of the tank (Fig 2D) and swam more slowly with shorter distances (Fig 2E) than their unaffected siblings. In addition, reproductive capacity was significantly lower in affected mutants compared to unaffected mutants, leading to an overall decrease in reproduction in the entire group of mutant adults compared with age-matched wild-type animals (Fig 2F).

Interestingly, however, no defects in locomotion were observed in the mutant embryos during the hatching stage (i.e., within 7 dpf). Both wild-type and mutant embryos responded rapidly when touched on the tail (S3 and S4 Movies). We hypothesized that mutant embryos do not exhibit behavioral defects because they have not yet developed excess Mn levels, as shown by our ICP-MS results (Fig 1E); moreover, during this stage, excess metals are not imported, as they have not begun feeding. In humans, manganism gives rise to a well-defined movement disorder with symptoms resembling Parkinson's disease [34,35]. Therefore, we exposed mutant embryos to various concentrations of Mn^2+^ at specific developmental stages in order to induce manganism.

Wild-type embryos have robust *slc30a10* expression starting at 5 dpf (Fig 1B), which suggests the start of a Mn-sensitive stage in development. We therefore exposed both wild-type and mutant embryos to various concentrations of Mn^2+^ at 5 dpf and analyzed the embryos at 6 dpf. At 300 μM, Mn caused a clear color change in the brain and liver of mutant embryos, but had no effect in wild-type embryos (Fig 2G). We then measured Mn levels in both Mn-treated and untreated wild-type and mutant embryos and found more accumulated Mn in mutants compared to wild-type (Fig 2H). Moreover, this accumulation in the mutant embryos persisted after the animals were transferred to Mn-free Holt buffer, suggesting that *slc30a10* plays an important role in the clearance of accumulated Mn. Finally, following exposure to relatively low concentrations of Mn^2+^ (150–200 μM), mutant embryos—but not wild-type embryos—had impaired locomotion and other defects, including tremor, postural instability, disorientation, and impaired balance (Fig 2I and 2J; S5 and S6 Movies). At higher concentrations of Mn^2+^ (250–300 μM), the mutants developed more severe defects, including body distortion, rigidity, bradykinesia, and a lack of escape response (Fig 2I and 2J; S7–S10 Movies). In addition, mutant embryos exposed to 300 μM Mn swam significantly shorter distances than both wild-type embryos and untreated mutant controls (Fig 2K). At even higher doses (>300 μM), Mn was lethal to some of the mutant animals.

GABAergic neurons are the primary target affected by Mn toxicity [21]. We therefore measured the expression of GABA transporters, GABA receptors, and the glutamic acid decarboxylases Gad65 and Gad67 in Mn-treated and untreated embryos. Exposure to Mn significantly reduced the expression of the GABA transporter *slc32a1* in both wild-type and mutant embryos (Fig 2L); in contrast, expression of the GABA receptors *gabbr1a* and *gabbr1b* was reduced only in the Mn-treated mutants (Fig 2M and 2N). Finally, Gad65/67 protein levels were significantly increased in Mn-treated wild-type embryos, as well as in both untreated and treated mutants (Fig 2O). Taken together, the reduced expression of the GABA transporter and receptors indicates reduced GABA uptake, while increased glutamic acid decarboxylases suggest more GABA production. These results suggest that extracellular GABA could be increased in both mutant embryos and Mn-treated embryos.

Impaired dopaminergic signaling has been attributed to the parkinsonism-like effects associated with Mn accumulation in the basal ganglia of both HMDPC patients and workers with occupational Mn exposure [8,12,23,35]. Zebrafish models of Parkinson's disease develop two classic pathological features, namely locomotor deficits and a loss of dopaminergic neurons [36]. Moreover, the escape response in zebrafish embryos requires functional dopaminergic circuits [37,38]. We therefore investigated whether the Mn-induced parkinsonism-like phenotype observed in Mn-exposed mutant embryos is due to a defect in dopaminergic signaling by measuring the expression of the dopamine transporter (*dat*) and tyrosine hydroxylase (*th*) in embryos with and without Mn exposure; both *dat* and *th* mRNA are commonly measured as molecular markers of dopaminergic neurons in zebrafish [39,40]. We found that under basal conditions, both *dat* and *th* gene expression patterns were similar between mutant and wild-type embryos. However, following exposure to Mn, mutant embryos had significantly lower levels of both *dat* and *th* mRNA (Fig 2P and 2Q; S3C and S3D Fig), suggesting decreased dopaminergic signaling. In addition, Mn-treated mutants—but not wild-type or untreated mutant embryos—had increased apoptosis markers in the brain (Fig 2R; S3E and S3F Fig), consistent with neuronal death specifically in mutant embryos following Mn exposure. Taken together, these data suggest that Mn accumulation leads to impaired GABAergic transmission and loss of dopaminergic neurons, possibly explaining the impaired locomotion observed in mutant zebrafish with Mn accumulation.

### *Slc30a10* mutant zebrafish develop hepatic pathology

**Fig 3:**
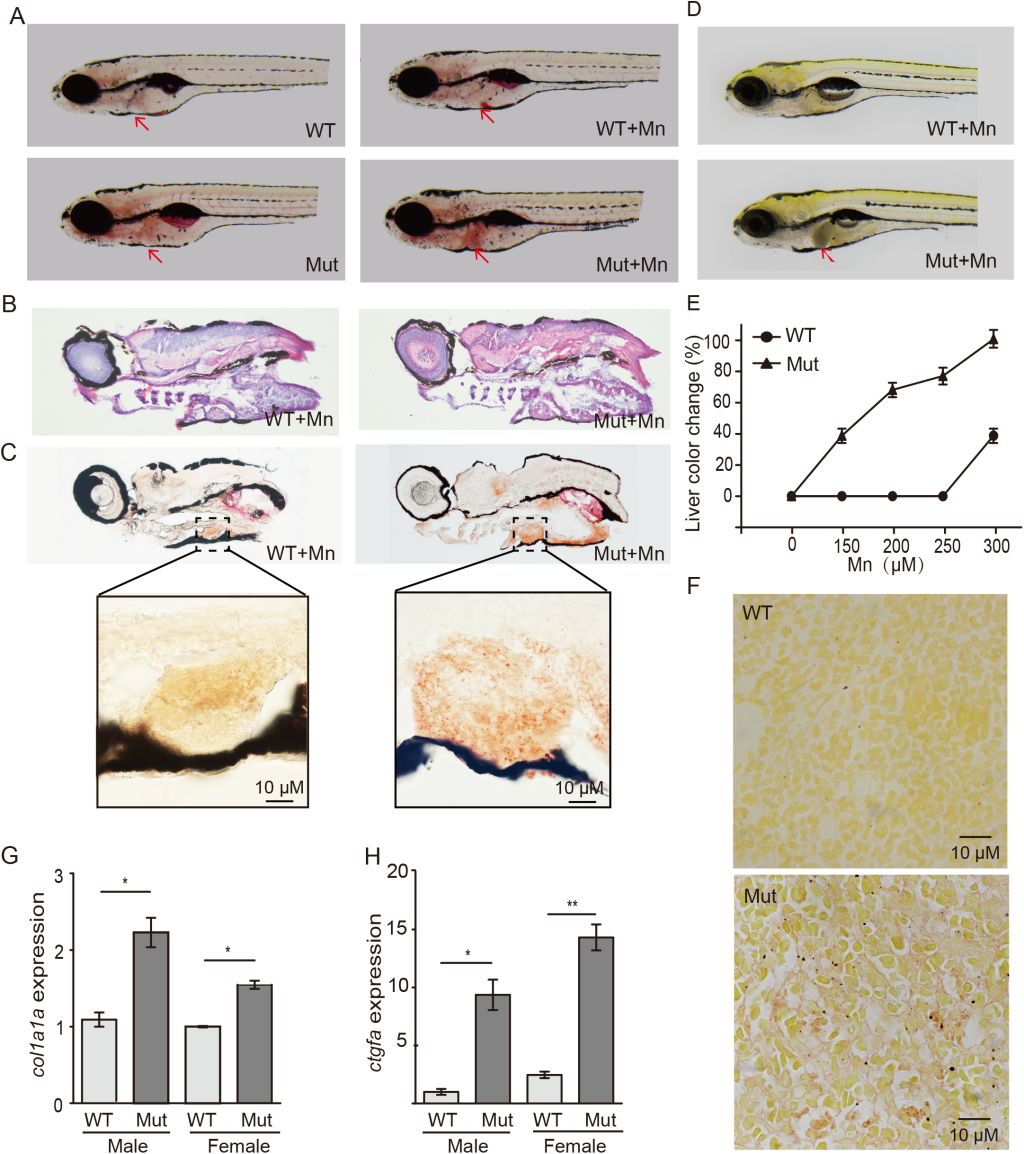
*Slc30a10* mutant zebrafish develop liver damage. (A) Oil red O staining shows that mutant embryos have a slightly fatty liver (red arrows), which was worsened upon exposure to Mn. (B-C) HE staining (B) and Oil red O staining (C) of frozen sections showing hepatic steatosis in mutant embryos following Mn exposure. (D) Example images of a wild-type and mutant embryo under Mn exposure, showing a darker colored liver in the mutant (red arrow). (E) Dose-response curve showing the percentage of embryos with liver color change versus Mn concentration (n = 3 sets of 20 embryos/group). (F) Image of the liver of a wild-type and mutant adult, showing severe fibrosis in the mutant liver with sirius red staining. (G-H) The fibrosis markers *col1a1a* and *ctgfa* were measured in the liver of both male and female WT and mutant animals (n = 3 sets of 20 adults/group; *p<0.05 and **p<0.01).

Patients with HMDPC develop a wide range of liver complications, including steatosis, hepatomegaly, hepatic fibrosis, and—ultimately—cirrhosis [23,26]. We therefore examined whether our mutant zebrafish develop a similar hepatic phenotype. Under basal conditions, mutant embryos develop a mild fatty liver, and this effect was increased upon Mn exposure (Fig 3A). HE staining and Oil red O staining of frozen sections confirmed hepatic steatosis in Mn-exposed mutant embryos (Fig 3B and 3C). In addition, Mn exposure caused a dose-dependent darkening of the liver in mutant embryos (Fig 3D and 3E), consistent with hepatic Mn accumulation. At 4 months of age, Sirius red staining revealed hepatic fibrosis in mutant animals (Fig 3F). Moreover, the mRNA levels of the fibrosis biomarkers *col1a1a* and *ctgfa* were higher in mutant animals compared to wild-type animals (Fig 3G and 3H). These data indicate that *slc30a10* mutant zebrafish develop hepatic pathology similar to the symptoms associated with HMDPC.

### *Slc30a10* mutant zebrafish develop polycythemia

Polycythemia develops early in all patients with HMDPC and is clinically distinguishable from patients with environmentally induced manganism [22,23,26]. We therefore measured the relative number of erythrocytes in both wild-type and mutant zebrafish. To visualize the erythrocytes, the animals were crossed to the *Tg(globinLCR*:*eGFP)* background, in which erythrocytes are labeled with GFP [41]; we then measured erythrocytes using FACS analysis.

**Fig 4:**
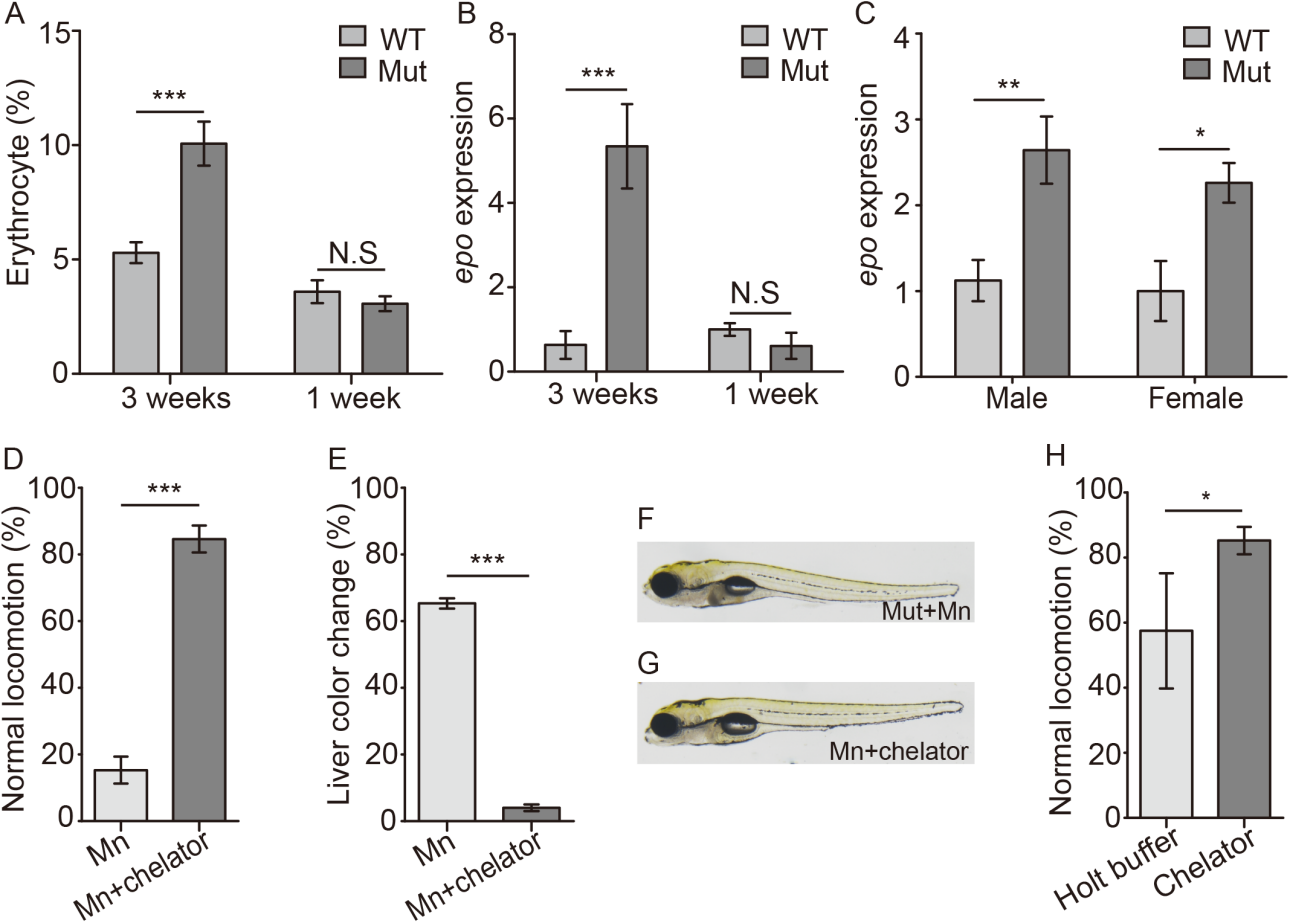
*Slc30a10* mutant zebrafish develop polycythemia, and chelation treatments improves the phenotype in mutant embryos. (A) Summary of the relative numbers of erythrocytes in 3-week-old and 1-week-old embryos (n = 6 sets of 50 embryos/group). (B and C) *Epo* expression was measured in 3-week-old and 1-week-old mutant zebrafish (B) and in the liver of adult zebrafish (C); n = 3 sets of 20 adults/group. (D and E) Mn-induced locomotor defects (D) and the darker color in liver (E) were rescued by treating embryos with EDTA-CaNa_2_ (chelator, n = 3 sets of 20 embryos/group). (F-G) Example images of a Mn-exposed mutant embryo, showing a darker colored liver (F), and a Mn-exposed mutant embryo following EDTA-CaNa_2_ treatment (G). (H) Following exposure to 300 μM Mn, mutant embryos were transferred to either Holt buffer alone or Holt buffer containing EDTA-CaNa_2_, and locomotion was measured (n = 3 sets of 20 embryos/group). *p<0.05, **p<0.01, and ***p<0.001.

Our analysis revealed an increase in GFP-positive cells in 3-week-old mutants compared with wild-type animals (Fig 4A), consistent with polycythemia. Increased systemic Mn may drive erythropoiesis by increasing the expression of erythropoietin (Epo), similar to hypoxia-stimulated erythropoietin expression [42]. We therefore measured *epo* expression in 3-week-old animals and found significantly increased expression in mutant animals (Fig 4B), and increased hepatic *epo* in 4-month-old mutants (Fig 4C). Interestingly, however, at the 1-week-old embryonic stage, neither the number of erythrocytes nor the expression of *epo* differed between mutant and wild-type embryos (Fig 4A and 4B). These results suggest that the polycythemia in mutant embryos develops secondary to the chronic accumulation of Mn and does not arise directly from *slc30a10* deficiency.

### EDTA-CaNa_2_ treatment partially rescues the phenotype in s*lc30a10* mutant zebrafish

Chelation therapy with EDTA-CaNa_2_ is commonly used to treat manganism [18,23]. We therefore tested whether chelation therapy can prevent the Mn-induced phenotype in our mutant zebrafish model by exposing mutant embryos to Mn in the presence or absence of EDTA-CaNa_2_. Treating mutant animals with EDTA-CaNa_2_ restored both locomotion and the escape response (Fig 4D and S11 Movies), and it reduced the Mn-induced hepatic color change (Fig 4E–4G), suggesting that EDTA-CaNa_2_ chelation is an effective treatment for *slc30a10* mutant zebrafish and that this model may be used to screen other chelation therapies. To test whether chelation therapy can reverse the effects of Mn (thereby mimicking better the therapeutic approach in patients), we first exposed mutant embryos to Mn; affected embryos with impaired locomotion and hepatic pathology were then transferred to Mn-free Holt buffer with or without EDTA-CaNa_2_ (2 mg/ml). Treatment with EDTA-CaNa_2_ improved the locomotion of Mn-exposed mutant embryos (Fig 4H), but had no effect on hepatic color change (S3G Fig), indicating that this therapy partially reverses the Mn-induced phenotype in *slc30a10* mutant zebrafish.

### Ferrous fumarate partially rescues the phenotype in s*lc30a10* mutant zebrafish

Ferrous fumarate has been used to reduce intestinal Mn absorption and relieve hypermanganesemia in patients with HMDPC [22,28,29]. Therefore, we exposed mutant embryos to Mn in the presence or absence of ferrous fumarate at 5 dpf and examined their phenotype the following day. Ferrous fumarate treatment caused the gut to turn brown in color, which suggest increased iron uptake in these embryos (S3H Fig). Importantly, however, treating mutant embryos with ferrous fumarate significantly improved locomotion (S3I Fig) and significantly reduced the rate of hepatic color change (S3J Fig), consistent with the notion that iron competes with Mn for intestinal import and can partially rescue Mn-induced hypermanganesemia in zebrafish embryos.

### Increased expression of *atp2c1* (*spca1*) in *slc30a10* mutant zebrafish

**Fig 5:**
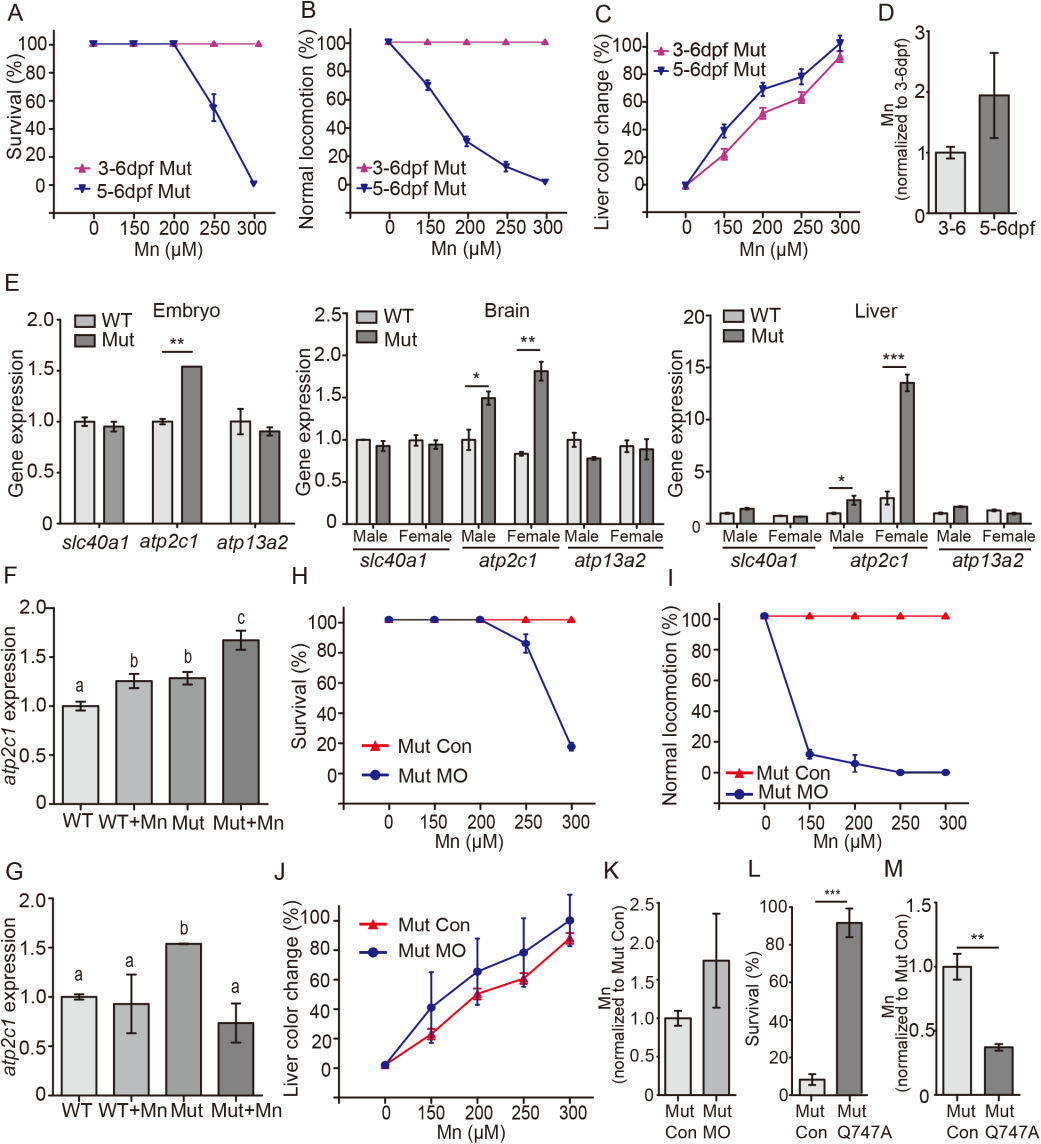
*atp2c1* expression is increased in *slc30a10* mutant zebrafish. (A-C) Mutant embryos were exposed to the indicated concentration of Mn for 24 h starting at 5 dpf (“5-6dpf” group) or 72 h starting at 3 dpf (“3-6dpf” group). At 6 dpf, survival (A), locomotion (B), and dark colored live (C) were measured. (D) Summary of normalized Mn levels in 3–6 dpf and 5–6 dpf mutants. (E) *Slc40a1*, *atp2c1*, and *atp132a* mRNA was measured in WT and mutant embryos (left), as well as in the liver (middle) and brain (right) of WT and mutant adults. (F-G) Summary of *atp2c1* expression measured in the 3–6 dpf group (F) and in the 5–6 dpf group (G); groups with different letters differed significantly (*p*<0.05). (H-J) Mutant embryos were injected with an *atp2c1* morpholino (Mut MO) or a scrambled control (Mut Con), the embryos were exposed to the indicated concentration of Mn for 72 h starting at 3 dpf, and survival (H), locomotion (I), and liver color change (J) were measured. (K) Summary of normalized Mn concentration in Mn-exposed Mut MO and Mut Con embryos. (L) Mutant embryos were injected with an mRNA encoding human ATP2C1 containing the Q747A mutation (Mut Q747A) or the control, and 3 dpf embryos were exposed to 2 mM Mn. (M) Embryos injected with Mut Q747A mRNA have reduced Mn accumulation than control mutants following Mn exposure. A-C, E-J, and L: n = 3 sets of 20 animals/group; D, K, and M: n = 3 sets of 200 embryos/group. *p<0.05, **p<0.01, and ***p<0.001.

In preliminary experiments, we found that exposing mutant embryos to 300 μM Mn at 2–3 dpf (prior to the onset of s*lc30a10* expression) had no effect, even when the embryos were exposed for several days; in contrast, the same concentration of Mn is toxic to slightly older (i.e., 5–6 dpf) mutant embryos. We therefore exposed mutant embryos to 300 μM Mn for 72 hours beginning at 3 dpf (“3–6 dpf mutants”) or for 24 hours beginning at 5 dpf (“5–6 dpf mutants”); we then examined the phenotype of both groups at 6 dpf. We found that embryos exposed beginning at 5 dpf had a clear phenotype, including reduced survival rate, locomotion defects and a change in liver color (Fig 5A–5C; S12 Movies). Strikingly, however, embryos there were exposed to Mn for a longer period (beginning at 3 dpf) were unaffected (Fig 5A–5C; S4A–S4C Fig). Using ICP-MS, we detected slightly lower Mn levels in 3–6 dpf mutants compared to 5–6 dpf mutants, despite a longer period of Mn exposure (Fig 5D). These data suggest that prior to the onset of *slc30a10* expression, another exporter protein may be expressed, thereby preventing the toxic effects of Mn exposure.

To test this idea, we measured the expression of several other exporters, including *atp2c1*, *slc40a1*, and *atp13a2* [43], and examined whether these genes are upregulated in *slc30a10* mutants. Our analysis revealed that only one gene—*atp2c1*—is upregulated in mutant embryos compared to wild-type animals (Fig 5E), suggesting that this gene may compensate for the dysfunction of *slc30a10*.

We also examined the effect of Mn exposure in 3 dpf wild-type and mutant embryos and found increased expression of *atp2c1* (Fig 5F)*—*but not *slc40a1* or *atp13a2* (S4D and S4E Fig)*—*in both wild-type and mutant embryos, suggesting that *atp2c1* is selectively upregulated in response to excess Mn at this early developmental stage. Interestingly, however, *atp2c1* expression was unaffected by Mn exposure in either wild-type or mutant embryos at 5 dpf (Fig 5G; S4F and S4G Fig), suggesting the presence of a relatively narrow developmental window in which *atp2c1* expression can respond to Mn exposure. Taken together, these data indicate that although the expression of *atp2c1* is higher in *slc30a10* mutant embryos compared to wild-type embryos under basal Mn conditions, *atp2c1* expression responds to Mn exposure only in an early developmental stage, suggesting that Atp2c1 may play a compensatory role as an Mn exporter when *slc30a10* expression is low.

To test the notion that Atp2c1 can functionally replace Slc30a10 in early development, we measured expression patterns using *in situ* hybridization and found that *atp2c1* is expressed in the brain and abdominal viscera of zebrafish embryos (S4H Fig), which overlaps partially with the expression pattern of *slc30a10* (i.e., in the brain and liver), indicating that *atp2c1* and *slc30a10* are expressed in similar organs. Next, we used a morpholino to block *atp2c1* expression in wild-type embryos and examined the effects of 72 hours of Mn exposure beginning at 3 dpf. Although Mn-treated WT-MO embryos survived (S4I Fig), they had impaired locomotion and developed a dark-colored liver; in contrast, wild-type embryos injected with a control (scrambled) morpholino were unaffected by Mn exposure (S4J and S4K Fig). These data suggest that Atp2c1 plays a protective role against Mn toxicity in early zebrafish development.

Next, we examined the effect of blocking *atp2c1* expression in *slc30a10* mutant embryos exposed to Mn for 72 hours beginning at 3 dpf. We found that MO-treated mutant embryos were more sensitive to Mn exposure and had reduced survival and more severely impaired locomotion compared to control mutants injected with a scrambled morpholino (Fig 5H and 5I). In addition, MO-injected mutant embryos had a darker colored liver (Fig 5J) and higher levels of Mn compared to control mutants (Fig 5K). Taken together, these data support the notion that Atp2c1 can function as a Mn exporter under systemic Mn stress and can protect *slc30a10* mutant embryos from Mn-induced toxicity selectively in early developmental stages.

Recently, Mukhopadhyay and Linstedt suggested that increasing the Mn-pumping capacity of ATP2C1 may be a feasible strategy for treating Mn overload in patients [44]. To test this strategy using our zebrafish model, we injected single-cell mutant embryos with human *ATP2C1* mRNA containing the gain-of-function Q747A mutation (ATP2C1-Q747A) and measured Mn clearance at 3 dpf. Compared with control mutant embryos, ATP2C1-Q747A‒injected embryos had higher Mn resistance and lower Mn levels (Fig 5L and 5M), confirming that overexpressing ATP2C1-Q747A improves Mn clearance in zebrafish embryos early in development. Interestingly, however, ATP2C1-Q747A‒injected mutants were not rescued when exposed to Mn at 5 dpf, possibly due to a degradation of mRNA by this stage.

### Atp2c1 can compensate for Slc30a10 in Mn metabolism

We found that although both 3–6 dpf and 5–6 dpf mutant embryos (i.e., mutant embryos exposed to Mn for 72 or 24 hours, respectively) developed a color change in the liver (Fig 5C), only the 5–6 dpf mutants had decreased survival and impaired locomotion (Fig 5A and 5B). We hypothesized that this difference could be due to a difference in the subcellular distribution of Mn between 3–6 dpf and 5–6 dpf mutants. Due to a lack of adequate antibodies against the zebrafish proteins, we tested this hypothesis in HeLa cells [21,44,45].

**Fig 6:**
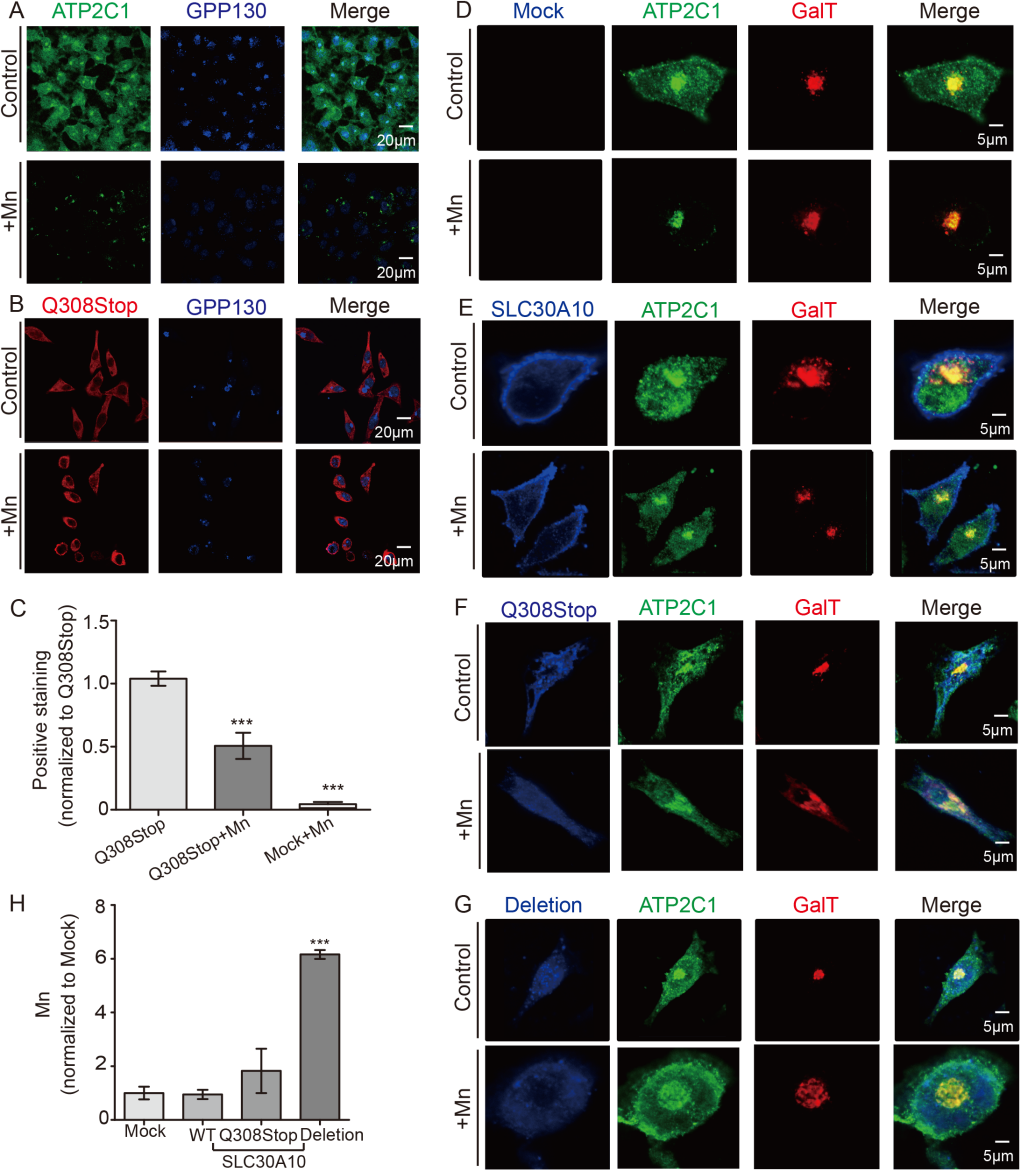
Functional crosstalk between SLC30A10 and ATP2C1 in HeLa cells. (A) HeLa cells were immunostained for ATP2C1 (green) and the Mn sensor protein GPP130 (blue). Following overnight exposure to 300 μM Mn, most of the ATP2C1 was translocated to the Golgi apparatus, and GPP130 was degraded. (B) HeLa cells were transfected with SLC30A10-Q308Stop-flag (Q308Stop, a mutant form of SLC30A10 in which codon 308 is changed to a stop codon); the cells were then immunostained for flag (red) and GPP130 (blue). Following Mn exposure, only half of the GPP130 was degraded. (C) Quantitative analysis of GPP130 immunoreactivity. (D) HeLa cells were transfected with galactosyltransferase-RFP (GalT) plasmid to label the Golgi apparatus (red); the cells were then immunostained for ATP2C1 (green). Following Mn exposure, most of the ATP2C1 was translocated to the Golgi apparatus, where it co-localized with GalT. (E-G) HeLa cells were co-transfected with the indicated constructs (E: GalT-RFP and SLC30A10-FLAG; F: GalT-RFP and SLC30A10-Q308Stop-flag; G: GalT-RFP and SLC30A10-1st-2nd-exons-deletion-flag, a mutant form of SLC30A10 in which the first and second exons are deleted); the cells were then immunostained for ATP2C1 (green) and FLAG (blue). (H) HeLa cells were mock-transfected cells or transfected with the indicated construct. After overnight exposure to Mn (500 μM), Mn concentration was measure using IPC-MS. ***p<0.001.

HeLa cells have barely detectable levels of *SLC30A10* expression and robust levels of *ATP2C1* expression (S5A Fig), which mimics the expression pattern of *slc30a10* and *atp2c1* in 3 dpf mutant zebrafish embryos (S5B Fig, 3 dpf). In contrast, expressing mutated *SLC30A10* in HeLa cells mimics the expression pattern of 5 dpf mutant zebrafish embryos (S5B Fig, 5 dpf). We then used GPP130, a Mn sensor, to measure intra-Golgi Mn levels [21,44]. We found that exposing control-transfected HeLa cells to Mn led to increased GPP130 degradation (i.e., increased Mn transport to the Golgi apparatus) (Fig 6A); in contrast, Mn transport to the Golgi apparatus was significantly decreased in HeLa cells transfected with the mutant *SLC30A10* (Fig 6B and 6C). Thus, by association we conclude that the subcellular distribution of Mn differs between 3–6 dpf mutants and 5–6 dpf mutants, possibly explaining their striking difference in phenotypes.

Next, we measured the subcellular localization of ATP2C1 in HeLa cells transfected with wild-type or mutant *SLC30A10*. We found that ATP2C1 was localized to the Golgi apparatus and throughout the cytoplasm under control conditions; however, following Mn treatment, the majority of ATP2C1 protein was localized to the Golgi apparatus (Fig 6A and 6D; S5C Fig). Wild-type SLC30A10 was located at the cell membrane (S5D Fig), whereas the mutant SLC30A10 was localized to the endoplasmic reticulum (S5D Fig). However, despite their differences in subcellular localization, both wild-type and mutant SLC30A10 disrupted the Mn-induced trafficking of ATP2C1 to the Golgi apparatus (Fig 6E–6G).

Next, we measured the levels of ATP2C1 protein in HeLa cells. We found that Mn exposure increased ATP2C1 expression in untransfected cells (S5F and S5G Fig), similar to our results in zebrafish embryos (Fig 5F). However, Mn exposure had no effect on ATP2C1 levels in HeLa cells transfected with wild-type SLC30A10 and slightly decreased ATP2C1 levels in HeLa cells transfected with mutant SLC30A10 (S5F and S5H Fig), similar to the expression pattern measured in zebrafish embryos at 5 dpf (Fig 5G). Taken together, these data indicate that ATP2C1 expression increases in response to Mn only when SLC30A10 expression is low.

Lastly, we measured Mn levels in HeLa cells following Mn exposure. We found that cells expressing mutant SLC30A10 accumulated significantly more Mn compared to control-transfected cells and cells expressing wild-type SLC30A10 (Fig 6H).

## Discussion

Here, we report the generation and characterization of two *slc30a10* mutant zebrafish lines. Our analysis shows that these mutant animals develop a phenotype strikingly similar to HMDPC in patients with a mutation in the *SLC30A10* gene, showing that the functional effects of these mutations are conserved between humans and zebrafish.

In wild-type zebrafish embryos, *slc30a10* is expressed in the brain and liver, and its expression is sensitive to Mn exposure, confirming that this transporter protein functions as a Mn exporter in vertebrates. Bakthavatsalam *et al*. recently used wild-type zebrafish embryos to study manganism [46]; however, their goal was to model locomotor defects induced by environmental and occupational exposure to high concentrations of Mn. Using a mutant zebrafish model, we found that *slc30a10* mutants have increased Mn sensitivity in the brain and liver. This approach enabled us to induce a phenotype using a relatively low concentration of Mn, thereby avoiding systemic Mn‒induced toxicity in wild-type animals. The manganism-like phenotype was reversed in wild-type embryos by removing the embryos from Mn-containing medium; however, Mn-exposed *slc30a10* mutant embryos were only partially rescued, thereby enabling us to investigate the underlying mechanisms.

Recently, Tuschl *et al*. reported that *slc39a14* mutant zebrafish are sensitive to Mn exposure and develop abnormal activity [17]; however, unlike the human ortholog, zebrafish *slc39a14* is not expressed in the liver [17], suggesting that *slc39a14* functions differently between zebrafish and humans. Although both *slc39a14* and *slc30a10* mutants accumulate Mn, only our *slc30a10* mutants have morphological and developmental defects, suggesting that *slc30a10* has a distinct function in regulating Mn homeostasis during zebrafish development. Interestingly, Hutchens et al. recently reported that *Slc30a10*-knockout mice have elevated systemic Mn levels [47]. It is important to note, however, that the severe hypothyroidism induced by Mn accumulation in *Slc30a10*-knockout mice differs from the symptoms present in patients with HMDPC. Therefore, we believe that our *slc30a10* mutant zebrafish, which fully recapitulate the symptoms of HMDPC in patients, may serve as a more suitable model.

It is interesting to note that in the embryonic stage, the mutant zebrafish have less Mn compared to wild-type embryos. During the pre-feeding embryonic stage, the embryo absorbs nutrients—including metals—primarily from the yolk via a process mediated by the yolk syncytial layer (YSL), an extra-embryonic syncytium that functions throughout early development to hydrolyze yolk materials and transport these materials from the yolk to the embryo’s cells and tissues [48,49]. Blocking the transport of nutrients from the YSL to the embryo causes nutritional deficiency. For example, Slc40a1 (ferroportin) is an iron exporter expressed in the YSL, and *slc40a1* mutant zebrafish embryos develop hypochromic anemia and iron deficiency [50]. Similar to *slc40a1*, *slc30a10* is also expressed in the YSL during early development (see Fig 1A), and mutating *slc30a10* could block the absorption of Mn from the yolk to the embryo, thus causing a Mn-deficient phenotype in mutant embryos. However, we found increased Mn accumulation in mutant animals during development from embryo to adult, suggesting that Slc30a10 has an extremely high affinity for Mn. The increase of Zn was comparable between the wild-type and mutants. Interestingly, Zn levels were slightly higher in mutant males than in wild-type males. Based on previous reports [33,51–53], it is possible that Slc30a10 might have an affinity for Zn in males. With respect to Fe, *Slc30a10*-knockout mice have increased Fe levels in the brain and blood in early developmental stages [47]; in contrast, we found no difference in Fe levels between wild-type and mutant zebrafish.

Up until 1 week of age, the *slc30a10* mutant embryos showed no behavioral deficits; however, upon exposure to relatively low concentrations of Mn (i.e., 150–300 μM), these animals develop a series of locomotor deficits, indicating that Mn readily accumulates in mutant embryos. Moreover, similar to patients with HMDPC, mutant zebrafish develop locomotor deficits and impaired liver function without environmental Mn exposure; these effects begin to manifest at 4 months of age and are likely due to the accumulation of normal dietary Mn, which is similar to patients [26]. Given that some mutants developed a neurological phenotype earlier than others, and given that affected mutants have higher Mn levels than their unaffected siblings, this variability in phenotypes may be due largely to individual differences in Mn absorption.

In the brain, the primary target for Mn is the basal ganglia, in which a tight balance between inhibitory and excitatory pathways maintains normal function of the network [46]. The majority of neurons in the basal ganglia are GABAergic; however, studies of the effect of Mn on the GABAergic system have yielded contradictory results; some groups reported increased GABA levels, whereas others reported decreased GABA levels [54]. Here, we found increased protein levels of GAD65/67 in mutant zebrafish; thus, increased extracellular GABA might reflect increased neuronal inhibition and impaired dopamine release. In addition, dopamine synthesis at dopaminergic terminals requires the enzyme tyrosine hydroxylase (TH), and Mn accumulation in dopaminergic terminals is mediated by the dopamine transporter (DAT) [55]. We found that the expression of both *dat* and *th* is more sensitive to Mn exposure in *slc30a10* mutants than in wild-type animals, suggesting that impaired dopaminergic transmission may underlie the locomotion defects in Mn-exposed mutants.

A member of the P-type family of ATPases, ATP2C1 pumps Ca^2+^ and Mn^2+^ ions from the cytosol into the Golgi apparatus and therefore plays an important role in regulating cellular ion homeostasis [56,57]. Knocking down the expression of *atp2c1* in zebrafish embryos reduced the embryo’s tolerance to Mn exposure in early development. In addition, we found that ATP2C1 is localized throughout the cytoplasm in HeLa cells under basal conditions, whereas ATP2C1 is localized primarily in the Golgi apparatus upon Mn exposure. Taken together, these *in vivo* and in *vitro* data provide compelling evidence that ATP2C1 can function as a Mn exporter.

The increased expression of *atp2c1* in *slc30a10* mutant zebrafish suggests a compensatory role between these two genes. In addition, we found significantly higher levels of *atp2c1* expression in the brain and liver of female mutants compared to male mutants (Fig 5E), suggesting that female mutants may be more tolerant of Mn. Interestingly, the neurological phenotype is less prevalent in female mutants than in age-matched male mutants; however, the mechanisms that underlie this gender difference in Mn sensitivity are currently unknown.

**Fig 7:**
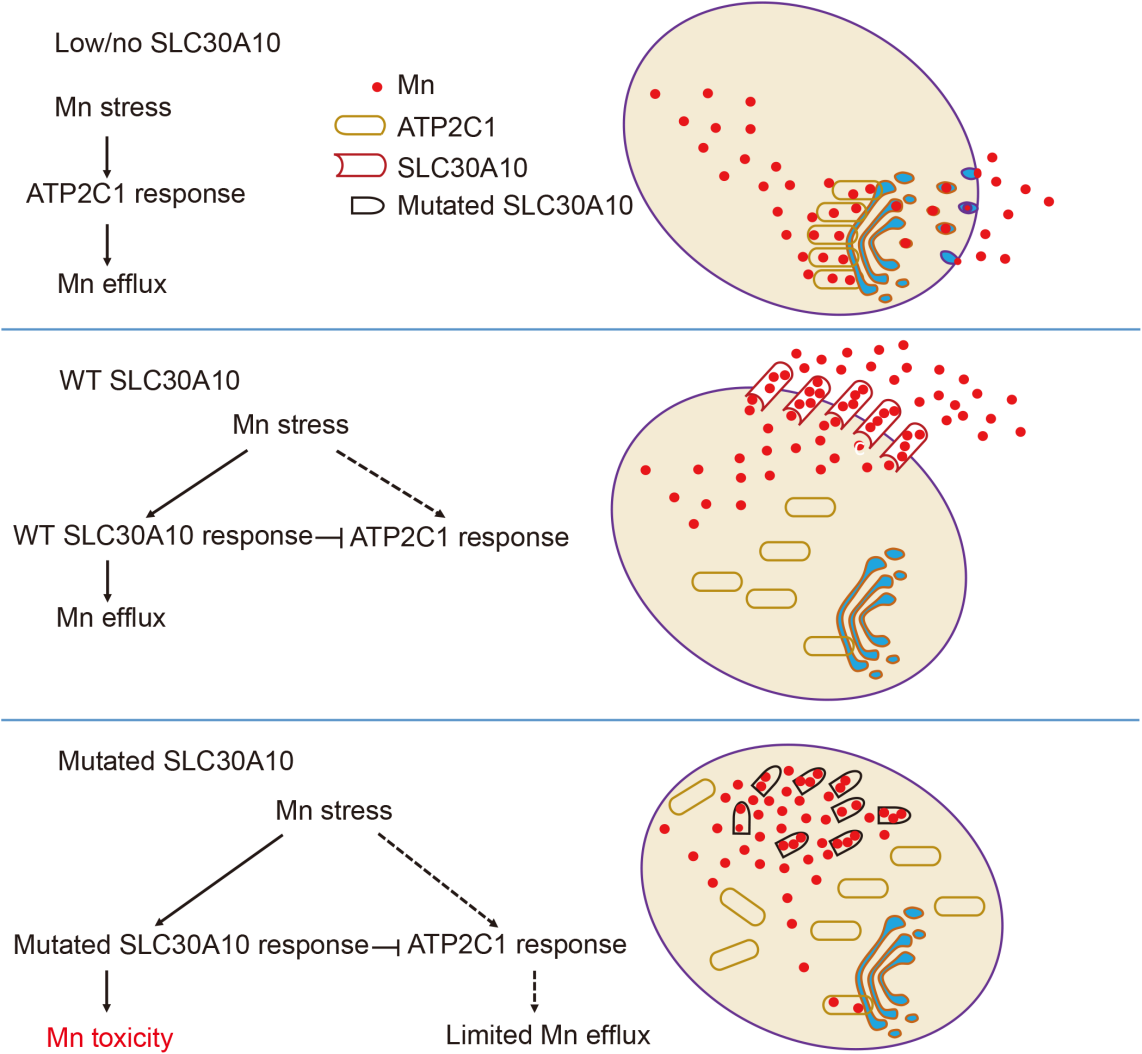
Schematic model depicting the proposed relationship between SLC30A10 and ATP2C1 in mediating Mn efflux. (Top) In the absence of SLC30A10, cells under Mn stress use ATP2C1 to clear Mn from the cytoplasm via Golgi vesicular trafficking. (Middle) When SLC30A10 levels are sufficient, cells under Mn stress use SLC30A10 as the primary Mn exporter, thereby bypassing the ATP2C1-mediated pathway. (Bottom) When cells express a mutant form of SLC30A10, Mn accumulates in the cytoplasm, causing Mn-induced toxicity; in addition, the ATP2C1-mediated pathway is reduced, limiting the efflux of Mn via Golgi vesicular trafficking.

Leyva-Illades *et al*. previously reported that HeLa cells expressing wild-type SLC30A10 have reduced Mn in the Golgi apparatus, as Mn efflux through SLC30A10 is both rapid and efficient [21]. Moreover, using DT40 cells (a chicken B cell line), Nishito *et al*. found that expressing SLC30A10 either alone or together with ATP2C1 induces similar levels of Mn resistance, suggesting that these two proteins are functionally redundant [58]. Our *in vivo* and *in vitro* results obtained with zebrafish embryos and HeLa cells, respectively, support the notion that ATP2C1 and SLC30A10 are functionally redundant with respect to exporting Mn from the cytoplasm; in addition, our results indicate that the effects of mutating SLC30A10 are mediated by regulating the expression of ATP2C1. Moreover, the subcellular redistribution of ATP2C1 upon Mn exposure is blocked by expressing either wild-type or mutant SLC30A10, and expressing mutant SLC30A10 leads to the accumulation of Mn in the cytoplasm (Fig 7). Interestingly, the mutant form of SLC30A10 (with both the first and second exons deleted) caused more accumulation of Mn in HeLa cells than the truncated form (with a premature stop codon at position 308) (Fig 6H), possibly due to the specific loss of the conserved amino acid that mediates Mn transport [23,45]. Taken together, these results suggest that upregulating ATP2C1 and/or restoring the Mn-responsive trafficking of ATP2C1 may be a viable strategy for increasing Mn clearance in cells in which SLC30A10 is dysfunctional, thereby providing new therapeutic options for patients with HMDPC. Nevertheless, precisely how SLC30A10 affects the Mn response and alters the trafficking of ATP2C1 is unknown and warrants investigation.

Patients with HMDPC develop impaired neurological function and liver damage, which can become life-threatening conditions. Therefore, given that our *slc30a10* mutant zebrafish develop impaired locomotion and liver damage due to systemic Mn accumulation following exposure to a relatively low dose of Mn, they represent a robust, reproducible model for studying these clinically severe complications. Because their skin is highly permeable, allowing the rapid absorption of compounds from the medium, zebrafish larvae are widely used for screening drugs. Using our *slc30a10* mutant embryos, we tested the therapeutic potential of EDTA-CaNa_2_ chelation and supplementation with ferrous fumarate, both of which are currently used in a clinical setting. Although EDTA-CaNa_2_ chelation had no effect on liver damage, it improved locomotor function, indicating that these zebrafish models are suitable for screening new chelating agents. Furthermore, ferrous fumarate supplementation partially alleviated the Mn-induced phenotype in our models. In addition, although chelators and ferrous fumarate are currently used for treating HMDPC patients, both treatments have side effects; EDTA chelation can induce changes in several other metals, including manganese, zinc, copper, and selenium, whereas ferrous fumarate can induce iron toxicity. Therefore, future research should focus on identifying chemical chaperones and/or other small compounds that specifically target Mn metabolism at the molecular level; in this respect, a drug that restores the Mn-responsive trafficking of ATP2C1 in cells with mutant SLC30A10 expression would be particularly relevant.

In conclusion, we developed and functionally characterized two independent lines of *slc30a10* mutant zebrafish and found that these models recapitulate the symptoms of patients with HMDPC, thereby providing an invaluable model for studying this genetic disorder. Mechanistically, we found that ATP2C1 plays a compensatory role in the context of SLC30A10 dysfunction, thereby maintaining Mn metabolism. These findings shed light on our understanding of the role that the Mn exporter SLC30A10 plays in the pathophysiology of HMDPC, and they provide experimental evidence for targeting ATP2C1 in managing HMDPC and related disorders.

## Materials and methods

### Mineral exposure

Zebrafish embryos were exposed to various concentrations of MnCl_2_ or ZnCl_2_, and their phenotypes and survival were recorded at different time points, depending on the specific experimental aims. Unless otherwise specified, the embryos were exposed to the indicated MnCl_2_ solution for 24 hours starting at 5 dpf (days post-fertilization).

### Reverse transcription and quantitative PCR

Total mRNA was isolated from the embryos and tissues using Trizol (Invitrogen). First-strand cDNA was synthesized using the PrimeScript RT reagent kit and Reverse Transcriptase M-MLV (M1701, Promega). Real-time PCR was performed using the two-step quantitative RT-PCR method with a kit (RR037A, Takara Bio), and target gene expression was normalized to *β-actin* mRNA levels.

### Whole-mount *in situ* hybridization

Whole-mount *in situ* hybridization was performed as described previously [59].

### Generation of knockout lines using CRISPR/Cas9

Mutant zebrafish lines were generated using previously reported methods [60]. In brief, a target site (GCTCTGCTTCTCCATCAGCATGG) in exon 1 of the *slc30a10* gene was selected, and the guide RNA (gRNA) template was amplified from the pMD-gata5-gRNA scaffold vector. *In vitro* transcription was performed using 1 μg of template DNA and T7 RNA polymerase. Zebrafish codon-optimized Cas9 plasmid [61] was linearized with *Xba*I, and Cas9-capped mRNA was transcribed using the mMESSAGE mMACHINE T7 kit (Ambion). The size and quality of the capped mRNA and gRNA were confirmed by electrophoresis through a 2% (w/v) agarose gel. Subsequently, 100 pg of gRNA and 400 pg of Cas9 mRNA were injected directly into single-cell embryos. The following primer pairs and the restriction enzyme *Bst*XI were used to assess the efficiency of genetic disruption: forward 5’-ACTCCTTCAACATGCTGTCGGACA-3’, reverse 5’-AGCACCTGTTTCCACCTGCT-3’. The injected embryos were raised to adulthood. F0 fish were crossed with wild-type fish to generate heterozygous F1 progeny, which were then genotyped using *Bst*XI digestion and DNA sequencing. Heterozygous F1 fish were crossed in order to generate homozygous mutant fish. The mutant and wild-type animals were obtained from heterozygous crosses.

### Inductively coupled plasma mass spectrometry (ICP-MS)

At least 1000 1-week-old embryos or 50 3-week-old larvae were collected in order to obtain the required amount of tissue (100 mg) for analysis. Single adult zebrafish were measured separately. At least three groups were measured for each stage. HeLa cells were seeded in 6-well dishes and transfected the next day. Twenty-four hours after transfection, the cells were exposed to 500 μM MnCl_2_ in serum-containing growth medium for 12 hours. After Mn exposure, the cells were washed with phosphate-buffered saline (PBS) and incubated in 500 μl trypsin-EDTA at 37°C for 3 min. The trypsin was then neutralized by adding 1 ml of serum-containing growth medium, and the cells were collected by centrifugation for 3 min in a microfuge. The supernatants were discarded, and the cell pellets were washed twice in 1 ml PBS containing 10 mM EDTA, followed by centrifugation as described above. After a final wash in PBS (without EDTA), the cell pellets were re-suspended in 200 μl RIPA buffer in order to extract the proteins; 1–2 μl of protein extract was used to measure protein concentration, and the remainder was used to measure the concentration of Mn. All samples were transferred to acid-washed cans with 5 ml metal-free HNO_3_ and 1 ml HF, then digested overnight at room temperature; the following day, 1 ml HClO_4_ was added, and the samples were incubated at 180°C for 10 hours. The cans were then transferred to 120°C and shaken for 2 hours in order to evaporate the residual HF and HClO_4_. Acid-washed vials were prepared by incubation in 20% HNO_3_ for 1 day, followed by extensive washing in ultrapure water. The digested samples were then diluted to 50 ml with Milli-Q water in the acid-washed vials and measured using an Agilent 7500 series inductively coupled plasma mass spectrometer.

### Western blot analysis

Whole embryos were lysed in modified RIPA buffer containing 150 mM sodium chloride, 50 mM Tris (pH 7.4), 1% Triton X-100, 1% sodium deoxycholate, 1% SDS, and proteasome inhibitor with PMSF, then centrifuged at 4°C for 15 minutes. Equal amounts of protein in each sample were resolved by SDS-PAGE on a 10% Mini-PROTEAN TGX Precast Gel (Bio-Rad) and transferred to an Immuno-Blot PVDF membrane (Bio-Rad). The membrane was blocked with 5% (w/v) non-fat milk and incubated with anti-GAD65/67 (ab11070, Abcam) and anti-actin (R1207-1, Huabio) antibodies. After washing three times for 10 minutes each with Tris-buffered saline containing Tween-20, the membranes were incubated with HRP-conjugated secondary antibodies for 1 hour at room temperature. Finally, the membranes were incubated in chemiluminescence reagent (34080, Pierce) and exposed to X-ray film.

### Phenotyping

Embryos were incubated in 6-well plates, and their morphological changes and behavioral patterns were observed and recorded using a Nikon SMZ18 stereo microscope. For measuring the escape response, a soft tip was used to touch the tail of embryo, and locomotion was recorded. For behavioral analysis, a digital video tracking system running the ANY-maze software program was used. The experiments were performed in a temperature-controlled room between 1:00 pm and 5:00 pm. The fish were allowed to acclimate to the system for 1 h before the start of data acquisition. Each adult fish was placed in a 20 cm × 10 cm tank containing 1 L of water, and the total distance traveled was recorded for 30 s. Each embryo was then placed in a well of a 24-well plate containing 2 ml Holt buffer with or without Mn, and the total distance traveled was recorded for 10 min.

### TUNEL apoptosis assay

Apoptosis were determined using the *in situ* Cell Death Detection Kit with TMR red (12156792910, Roche) in accordance with the manufacturer’s instructions. The fluorescence signal was analyzed using ImageJ software.

### Frozen sections

After fixation with 4% paraformaldehyde in PBS overnight, 6 dpf embryos were washed with PBS and incubated in a 30% sucrose solution overnight. The embryos were then incubated in a 1:1 mixture of 30% (w/v) sucrose and OCT compound (Tissue-Tek) for 2 hours, followed by overnight incubation in OCT. The samples were frozen in OCT compound at -80°C, and 10-μm cryo-sections were cut using a CM1950 cryostat (Leica).

### Immunofluorescence

Cells were fixed with 4% paraformaldehyde in PBS for 10 minutes, washed three times with PBS, and then permeabilized in 0.2% Triton X-100 in PBS for 5 minutes. After washing three times with PBS and blocking with PBS containing 5% fetal bovine serum (FBS), the cells were immunostained with rabbit anti-flag (2368s, Cell Signaling), rabbit anti-giantin (ab80864, Abcam), mouse anti-pan-Cadherin (ab22744, Abcam), or mouse anti-atp2c1 (WH0027032M1, Sigma) in PBS with 1% FBS at 4°C overnight. The cells were then washed three times with PBS and incubated with mouse anti-calnexin Alexa Fluor 647 (ab202572, Abcam), goat anti-rabbit Alexa Fluor 488 (A11008, Molecular Probes), donkey anti-mouse Alexa Fluor 488 (ab150105, Abcam), donkey anti-rabbit Alexa Fluor 555 (A31572, Molecular probes), and/or goat anti-rabbit Alexa Fluor 350 (A0408, Beyotime) in PBS with 1% FBS at 37°C for 1.5 hours. The slides were mounted under coverslips using ProLong Gold Antifade reagent (Thermo Fisher Scientific), and fluorescence images were obtained using an LSM-710 microscope (Zeiss, Germany) equipped with a 63X/1.4 numerical aperture oil-immersion objective.

### Cell culture and transfection

HeLa cells were grown in Dulbecco's modified Eagle's medium (DMEM, Gibco) supplemented with 10% FBS (Gibco) and 1X penicillin-streptomycin (Gibco). The cells were cultured at 37°C in humidified air containing 5% CO_2_. The cells were transfected using the X-tremeGENE HP DNA Transfection Reagent (Roche) in accordance with the manufacturer’s instructions. The cells were generally transfected 24 hours after plating and used 36 hours after transfection. Where indicated, freshly prepared MnCl_2_ solution was added to the medium at a final concentration of 300 μM for the indicated times.

### Oil red O staining

Embryos were fixed in 4% formaldehyde overnight at 4°C, washed with PBS, and stained with filtered 0.3% oil red O in 60% 2-propanol for 2 hours. After a final wash in PBS, the embryos were placed in depression wells and photographed.

### Erythrocyte measurements using FACS analysis

One-week-old and 3-week-old larvae on the *Tg(globinLCR*:*eGFP*) background were crushed in Hank's buffered salt solution (HBSS, Invitrogen), followed by complete dissociation to a single-cell suspension by trituration. The cells were washed and harvested in HBSS and then examined using a Cytomics FC 500 MCL flow cytometer (Beckman Coulter, Inc.).

### Microinjection of mRNA and morpholinos

The full-length wild-type human *ATP2C1* cDNA was cloned using PCR, and the Q747A mutation was introduced using two-step site-directed mutagenesis. The cDNA sequence containing the mutation was inserted into the pCS2 expression plasmid, linearized, and transcribed using the mMESSAGE mMACHINE SP6 kit (Ambion). The resulting mRNA was injected into one-cell stage zebrafish embryos at 200 pg/embryo. The *atp2c1* morpholino (5’-TGCATCTTCCGTCAGCTTTCGTTGC-3’) or a scrambled sequence was injected into one-cell stage embryos at 0.1 nmol/embryo.

### Rescue and recovery experiments

A final concentration of 2 mg/ml EDTA-CaNa_2_ was prepared and either mixed with MnCl_2_ (at a final concentration of 250–300 μM) or used alone. Saturated ferrous fumarate was added to 5 ml 200 μM MnCl_2_ to assess rescue efficiency. For controls, treated embryos were washed several times with fresh Holt buffer and then incubated in Holt buffer.

### Statistical analyses

All summary data are presented as the mean ± the standard error of the mean (SEM). Differences between two groups were analyzed using the two-tailed Student’s *t*-test, and multiple groups and time points were analyzed using a two-way ANOVA. Differences with a *p*-value <0.05 were considered significant.

### Ethics statement

Approval for the study was granted by the Institutional Animal Care and Use Committee of the Laboratory Animal Center, Zhejiang University. We followed the relevant guidelines from the Laboratory Animal Center of Zhejiang University. We anaesthetized or sacrificed the zebrafish by freezing them in ice before performing the experiments.

### Data availability

All relevant data are provided within the paper and the Supporting Information.

## Supporting information

S1 Fig Zebrafish embryos are a suitable model for studying Mn metabolism. (A) Wild-type embryos were analyzed at 6 dpf following Mn exposure at the indicated concentration for 24 hours, and the percentage of embryos that responded (defined as impaired locomotion) is plotted against Mn concentration. (B and C) Mn accumulates in wild-type embryos exposed to 1 mM Mn for 24 hours, shown as a dark color in the brain and liver (C, red arrows). (D) Summary of the fold change in Mn in the three body regions of wild-type embryos exposed to 1 mM Mn for 24 hours. (E) 24 hours after transferring an Mn-exposed embryo to fresh Holt buffer, the color in the brain and liver returned to basal levels. (F) Summary of the fold change in Mn accumulation in Mn-exposed embryos that were transferred to fresh Holt buffer. (G) RT-PCR of *slc30a10* mRNA in wild-type (+/+), heterozygous (+/-), homozygous 10-bp deletion (-10), and homozygous 1-bp insertion (+1) embryos. *β-actin* mRNA was measured as an internal control. (H) Predicted protein sequences of the wild-type (WT), -10, and +1 *slc30a10* alleles generated using CRISPR/Cas9-based editing.
(TIF)

S2 Fig SLC30A10 is conserved among vertebrates. Sequence alignment of the human, mouse, and zebrafish SLC30A10 proteins. Identical residues are indicated with an asterisk (*), highly conserved residues are indicated with a colon (:), and weakly conserved residues are indicated with a period (.).
(TIF)

S3 Fig Phenotypic characterization and effect of ferrous fumarate on *slc30a10* mutant embryos. (A) *Slc30a10* mRNA was measured in heterozygous adults with or without Mn exposure. (B) *Slc30a10* mRNA level was showed by a ratio of adults versus embryos in both wild-type and mutants. (C and D) Quantitative analyses of *in situ* staining for *dat* (C) and *th* (D) mRNA in WT and mutant embryos; where indicated, the embryos were exposed to Mn. (E) Quantitative analyses of TUNEL fluorescence. (F) HE staining in frozen sectioned brain of embryos. (G) Following Mn exposure, mutant embryos were transferred to either fresh Holt buffer of buffer containing the chelator EDTA-CaNa_2_, and the percentage of embryos with a color change in the liver is plotted (n = 3 sets of 20 embryos/group). (H) Image of a Mn-exposed mutant embryo after treatment with ferrous fumarate; note the brown color in the gut (arrow). (I and J) Summary of locomotion (I) and the percentage of embryos with a color change in the liver (J) in mutant Mn-exposed embryos treated with ferrous fumarate (n = 3 sets of 20 embryos/group). *p<0.05, **p<0.01, and ***p<0.001.
(TIF)

S4 Fig Atp2c1 functions as a Mn exporter in zebrafish embryos in early development. (A and B) Survival (A) and locomotor conditions (B) of wild-type embryos exposed to the indicated concentrations of Mn either for 72 h starting at day 3 dpf (3–6 dpf) or for 24 h starting at 5 dpf (5–6 dpf). (C) Percentage of embryos with a dark-colored liver after exposure to the indicated Mn concentrations. (D and E) *Atp13a2* (D) and *slc40a1* (E) mRNA levels were measured in wild-type and mutant embryos at 6 dpf; where indicated, the embryos were exposed to Mn at 3 dpf for 3 days. (F and G) *Atp13a2* (F) and *slc40a1* (G) mRNA levels were measured in wild-type and mutant embryos at 6 dpf; where indicated, the embryos were exposed to Mn at 5 dpf for 24 h. Groups with different letters differed significantly (*p*<0.05). (H) *In situ* hybridization for *atp2c1* mRNA in wild-type embryos at 3 dpf and 6 dpf. (I-K) Wild-type embryos were injected with an *atp2c1* morpholino (WT MO) or a scrambled morpholino (WT CON), then exposed to the indicated Mn concentration for 3 days at 3 dpf. (J and K) Injecting wild-type embryos with the *atp2c1* morpholino facilitates Mn-induced locomotion deficits (J) and dark colored liver (K).
(TIF)

S5 Fig Expression of ATP2C1 and SLC30A10 in HeLa cells. (A) HeLa cells express minimal *SLC30A10* and robust amounts of *ATP2C1*. (B) The expression of *slc30a10* and *atp2c1* in 3 dpf and 5 dpf embryos detected by RT-PCR. (C) HeLa cells were immunostained for ATP2C1 (green) and the endogenous Golgi markers GIANTIN (red). Following Mn exposure, most of the ATP2C1 proteins were translocated to the Golgi apparatus, where they co-localized with GALT. (D and E) HeLa cells were transfected with wild-type SLC30A10 (D), SLC30A10-Q308Stop (E, upper panels), or SLC30A10-Deletion (E, lower panels), then analyzed using immunofluorescence. Wild-type SLC30A10 co-localizes with Cadherin (measured using a pan-Cadherin antibody) at the cell membrane, whereas the mutant SLC30A10 co-localizes with Calnexin at the endoplasmic reticulum. (F-H) Western blot analysis of ATP2C1 (F) and quantification of the relative ATP2C1 protein levels in the indicated groups (G and H); groups with different letters differed significantly (*p*<0.05). HeLa cells were transfected with pCMV-wild-type-SLC30A10-flag (WT), pCMV-SLC30A10-Q308Stop-flag (Q308Stop), or pCMV-SLC30A10-1st-2nd-exons-deletion-flag (Deletion), and exogenous SLC30A10 expression was measured using a FLAG antibody.
(TIF)

S1 Table Metal levels in adult zebrafish tissue. (DOCX)

S1 Movie One mutant 6-month-old adult has locomotor disabilities, but its unaffected mutant sibling does not. (MOV)

S2 Movie One mutant 6-month-old adult has locomotor disabilities, but its unaffected mutant sibling does not. (MOV)

S3 Movie Wild-type embryos exhibit normal locomotion and a normal escape response at 6 dpf. (MOV)

S4 Movie Mutant embryos exhibit normal locomotion and a normal escape response at 6 dpf. (MOV)

S5 Movie After exposure to 150 μM Mn for 24 h at 5 dpf, some mutant embryos develop locomotor defects, loss of direction, and lack of balance at 6 dpf. (MOV)

S6 Movie After exposure to 150 μM Mn for 24 h at 5 dpf, mutants show tremor and postural instability at 6 dpf. (MOV)

S7 Movie After exposure to 250 μM Mn for 24 h at 5 dpf, wild-type embryos have normal locomotion and a normal escape response at 6 dpf. (MOV)

S8 Movie After exposure to 250 μM Mn for 24 h at 5 dpf, mutant embryos have severe locomotor defects at 6 dpf. (MOV)

S9 Movie After exposure to 250 μM Mn for 24 h at 5 dpf, mutant embryos have severe rigidity and tremor at 6 dpf. (MOV)

S10 Movie After exposure to 250 μM Mn for 24 h at 5 dpf, mutants have a lack of balance and impaired escape response at 6 dpf. (MOV)

S11 Movie Mutant embryos have normal locomotion and a normal escape response at 6 dpf after treatment for 24 h with both Mn and EDTA-CaNa_2_. (MOV)

S12 Movie Mutant embryos have normal locomotion and a normal escape response at 6 dpf after exposure to 250 μM Mn for 72 h at 3 dpf. (MOV)
