# Supplementary figures and images for: Zebrafish slc30a10 deficiency revealed a novel compensatory mechanism of Atp2c1 in maintaining manganese homeostasis
Source: PLoS Genet. 2017 Jul 10;13(7):e1006892. doi: 10.1371/journal.pgen.1006892 (PMC5524415; doi:10.1371/journal.pgen.1006892)

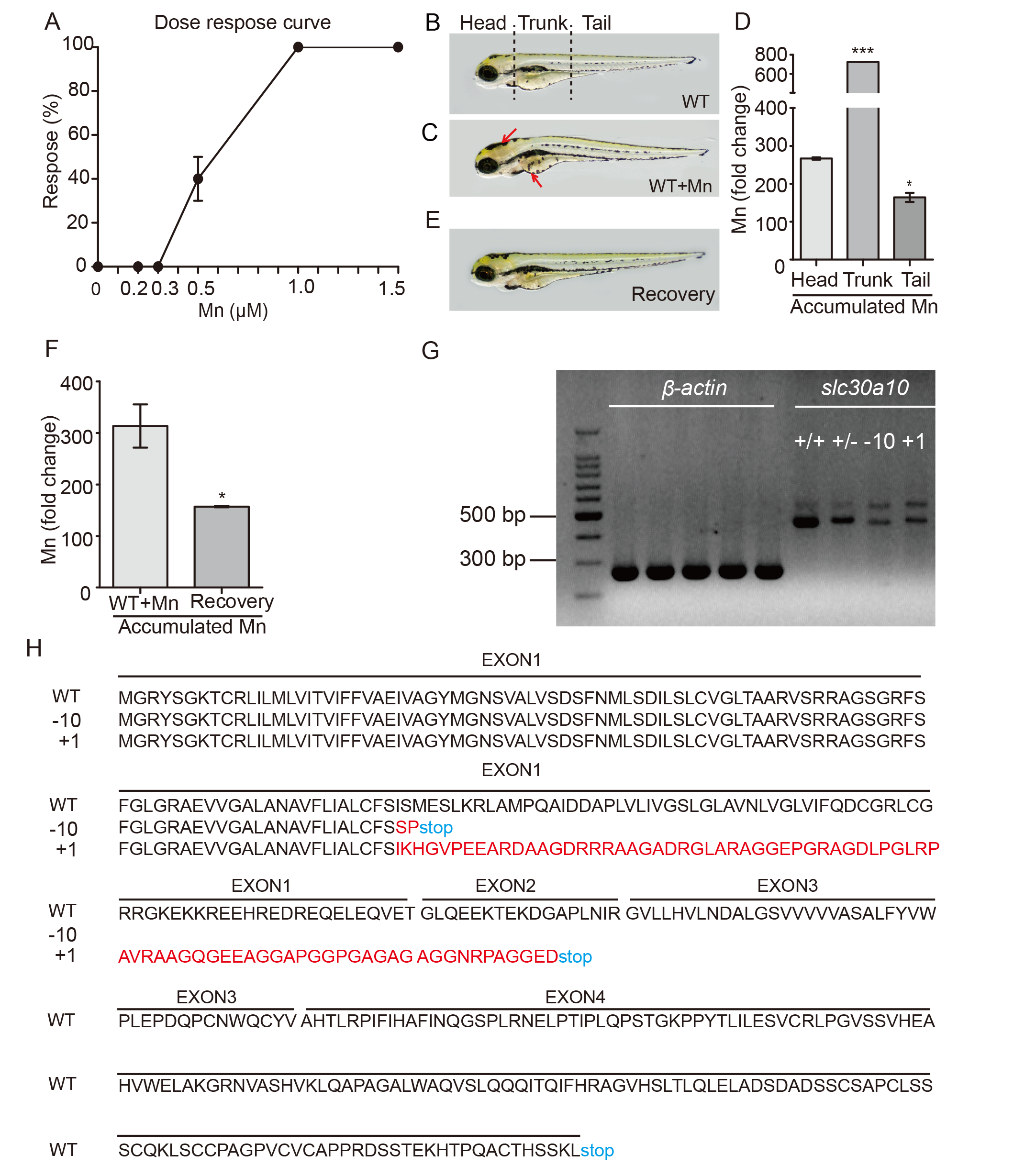

Supplement: S1 Fig — (A) Wild-type embryos were analyzed at 6 dpf following Mn exposure at the indicated concentration for 24 hours, and the percentage of embryos that responded (defined as impaired locomotion) is plotted against Mn concentration. (B and C) Mn accumulates in wild-type embryos exposed to 1 mM Mn for 24 hours, shown as a dark color in the brain and liver (C, red arrows). (D) Summary of the fold change in Mn in the three body regions of wild-type embryos exposed to 1 mM Mn for 24 hours. (E) 24 hours after transferring an Mn-exposed embryo to fresh Holt buffer, the color in the brain and liver returned to basal levels. (F) Summary of the fold change in Mn accumulation in Mn-exposed embryos that were transferred to fresh Holt buffer. (G) RT-PCR of slc30a10 mRNA in wild-type (+/+), heterozygous (+/-), homozygous 10-bp deletion (-10), and homozygous 1-bp insertion (+1) embryos. β-actin mRNA was measured as an internal control. (H) Predicted protein sequences of the wild-type (WT), -10, and +1 slc30a10 alleles generated using CRISPR/Cas9-based editing. (TIF) [file pgen.1006892.s001.tif]

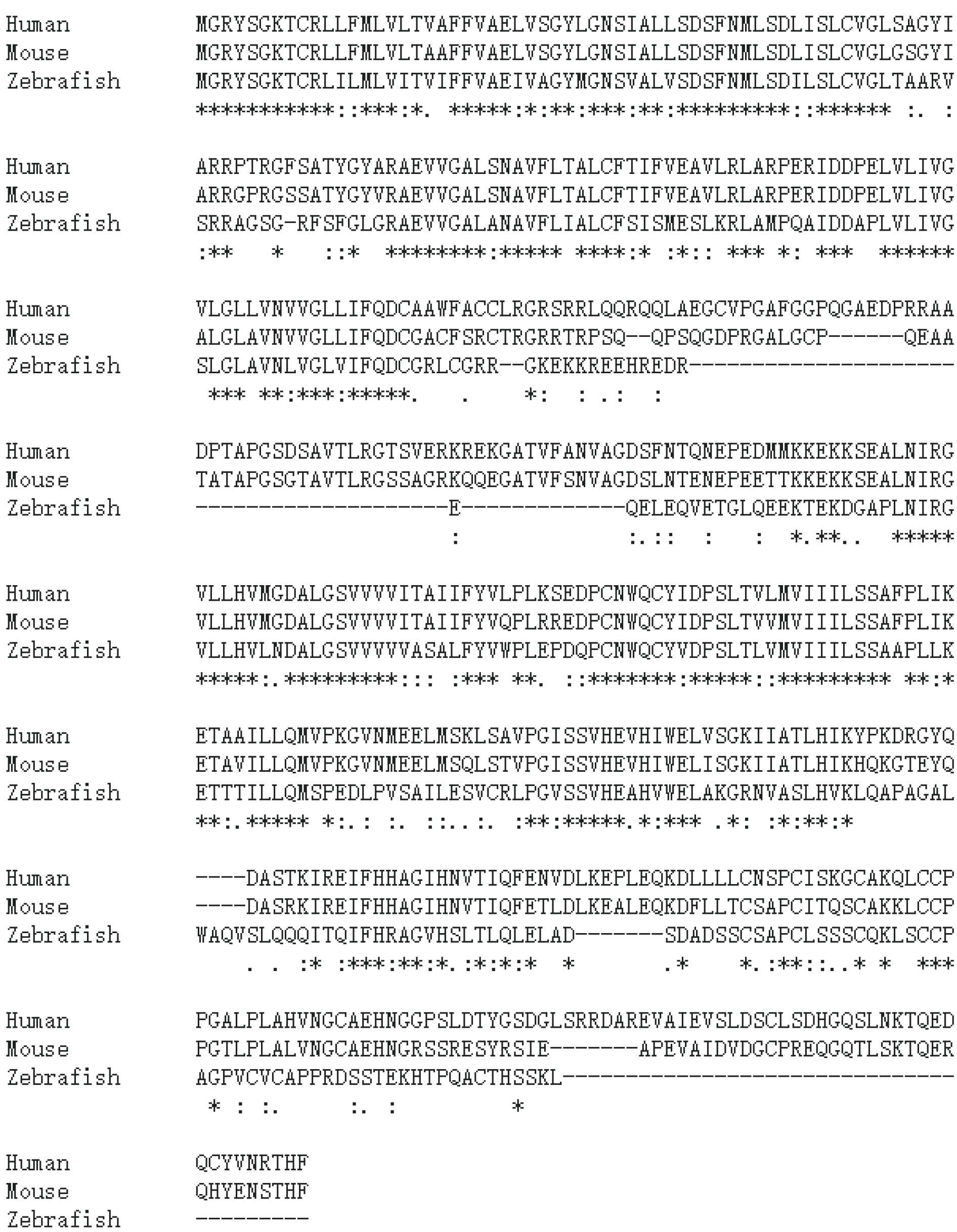

Supplement: S2 Fig — Sequence alignment of the human, mouse, and zebrafish SLC30A10 proteins. Identical residues are indicated with an asterisk (*), highly conserved residues are indicated with a colon (:), and weakly conserved residues are indicated with a period (.). (TIF) [file pgen.1006892.s002.tif]

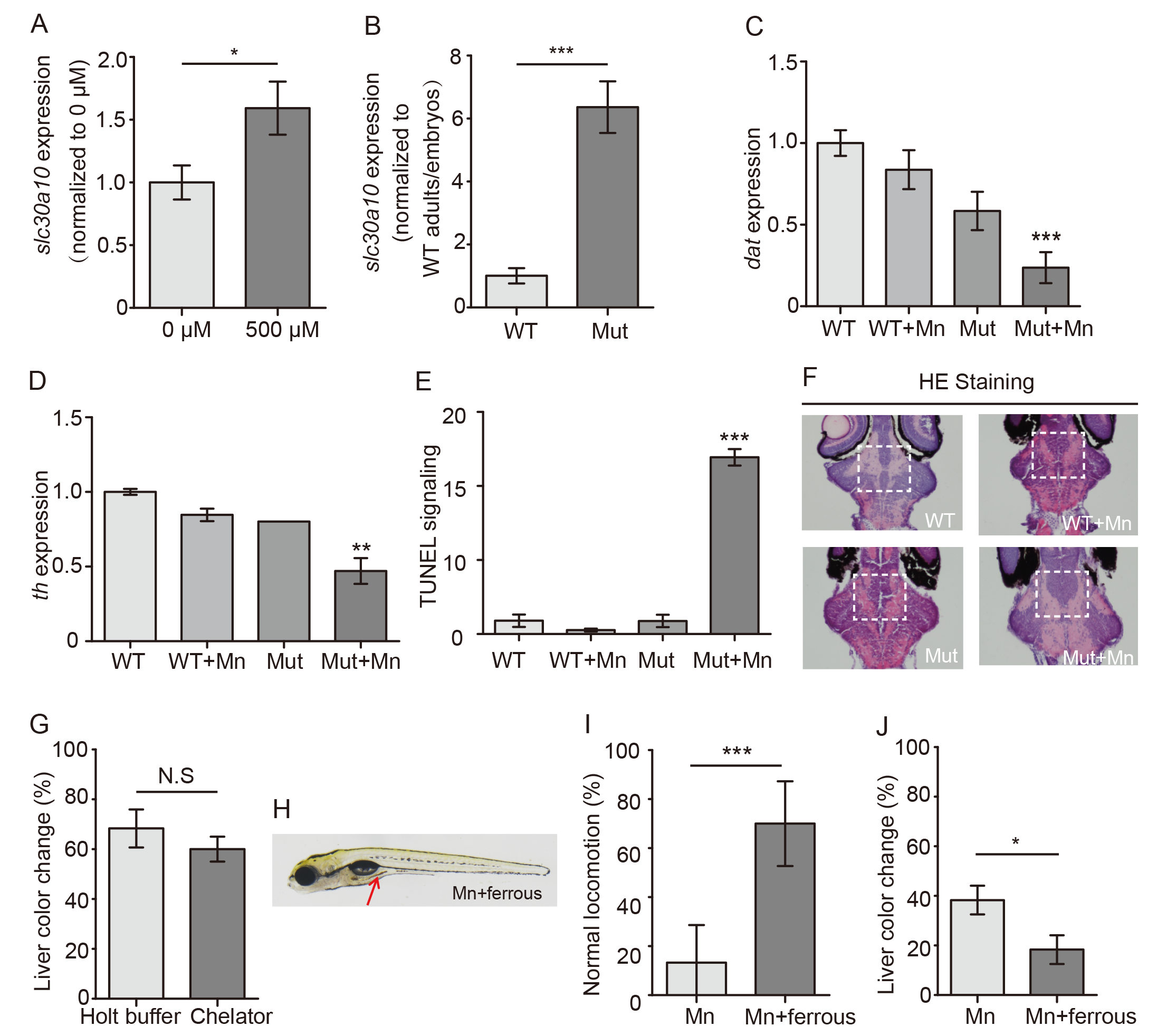

Supplement: S3 Fig — (A) Slc30a10 mRNA was measured in heterozygous adults with or without Mn exposure. (B) Slc30a10 mRNA level was showed by a ratio of adults versus embryos in both wild-type and mutants. (C and D) Quantitative analyses of in situ staining for dat (C) and th (D) mRNA in WT and mutant embryos; where indicated, the embryos were exposed to Mn. (E) Quantitative analyses of TUNEL fluorescence. (F) HE staining in frozen sectioned brain of embryos. (G) Following Mn exposure, mutant embryos were transferred to either fresh Holt buffer of buffer containing the chelator EDTA-CaNa2, and the percentage of embryos with a color change in the liver is plotted (n = 3 sets of 20 embryos/group). (H) Image of a Mn-exposed mutant embryo after treatment with ferrous fumarate; note the brown color in the gut (arrow). (I and J) Summary of locomotion (I) and the percentage of embryos with a color change in the liver (J) in mutant Mn-exposed embryos treated with ferrous fumarate (n = 3 sets of 20 embryos/group). *p<0.05, **p<0.01, and ***p<0.001. (TIF) [file pgen.1006892.s003.tif]

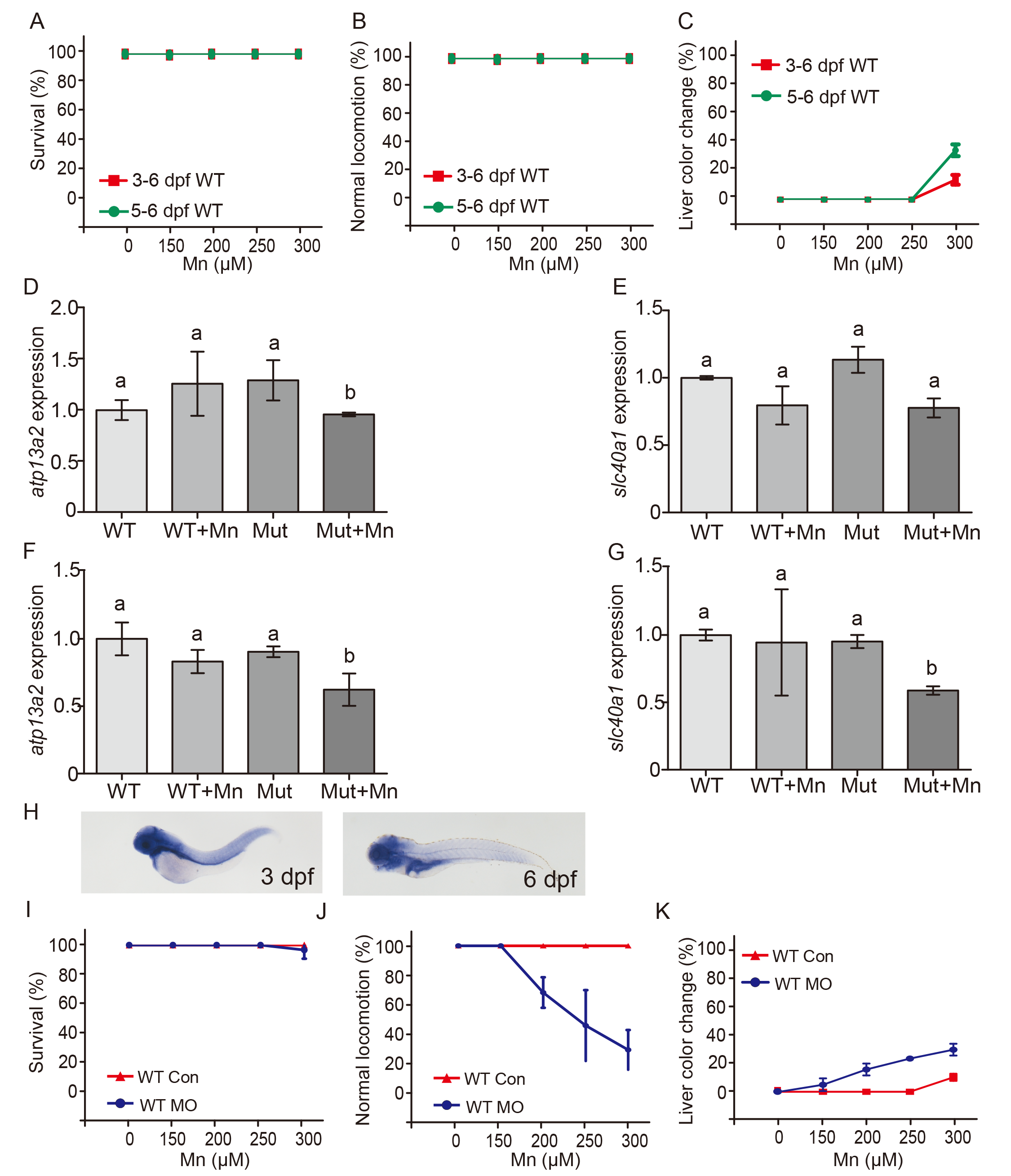

Supplement: S4 Fig — (A and B) Survival (A) and locomotor conditions (B) of wild-type embryos exposed to the indicated concentrations of Mn either for 72 h starting at day 3 dpf (3–6 dpf) or for 24 h starting at 5 dpf (5–6 dpf). (C) Percentage of embryos with a dark-colored liver after exposure to the indicated Mn concentrations. (D and E) Atp13a2 (D) and slc40a1 (E) mRNA levels were measured in wild-type and mutant embryos at 6 dpf; where indicated, the embryos were exposed to Mn at 3 dpf for 3 days. (F and G) Atp13a2 (F) and slc40a1 (G) mRNA levels were measured in wild-type and mutant embryos at 6 dpf; where indicated, the embryos were exposed to Mn at 5 dpf for 24 h. Groups with different letters differed significantly (p<0.05). (H) In situ hybridization for atp2c1 mRNA in wild-type embryos at 3 dpf and 6 dpf. (I-K) Wild-type embryos were injected with an atp2c1 morpholino (WT MO) or a scrambled morpholino (WT CON), then exposed to the indicated Mn concentration for 3 days at 3 dpf. (J and K) Injecting wild-type embryos with the atp2c1 morpholino facilitates Mn-induced locomotion deficits (J) and dark colored liver (K). (TIF) [file pgen.1006892.s004.tif]

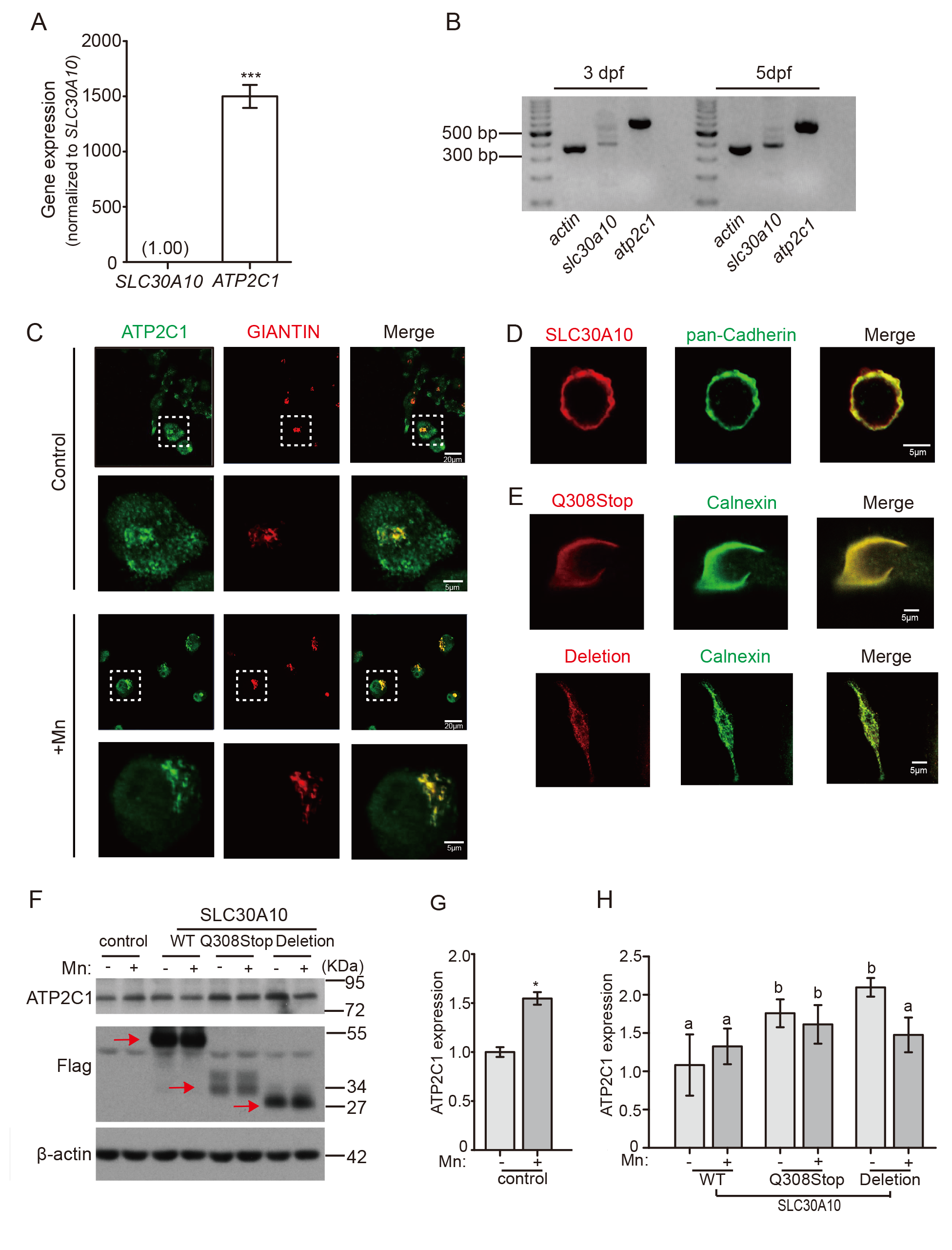

Supplement: S5 Fig — (A) HeLa cells express minimal SLC30A10 and robust amounts of ATP2C1. (B) The expression of slc30a10 and atp2c1 in 3 dpf and 5 dpf embryos detected by RT-PCR. (C) HeLa cells were immunostained for ATP2C1 (green) and the endogenous Golgi markers GIANTIN (red). Following Mn exposure, most of the ATP2C1 proteins were translocated to the Golgi apparatus, where they co-localized with GALT. (D and E) HeLa cells were transfected with wild-type SLC30A10 (D), SLC30A10-Q308Stop (E, upper panels), or SLC30A10-Deletion (E, lower panels), then analyzed using immunofluorescence. Wild-type SLC30A10 co-localizes with Cadherin (measured using a pan-Cadherin antibody) at the cell membrane, whereas the mutant SLC30A10 co-localizes with Calnexin at the endoplasmic reticulum. (F-H) Western blot analysis of ATP2C1 (F) and quantification of the relative ATP2C1 protein levels in the indicated groups (G and H); groups with different letters differed significantly (p<0.05). HeLa cells were transfected with pCMV-wild-type-SLC30A10-flag (WT), pCMV-SLC30A10-Q308Stop-flag (Q308Stop), or pCMV-SLC30A10-1st-2nd-exons-deletion-flag (Deletion), and exogenous SLC30A10 expression was measured using a FLAG antibody. (TIF) [file pgen.1006892.s005.tif]
