# Supplementary material for: Zebrafish slc30a10 deficiency revealed a novel compensatory mechanism of Atp2c1 in maintaining manganese homeostasis
Source: PLoS Genet. 2017 Jul 10;13(7):e1006892. doi: 10.1371/journal.pgen.1006892 (PMC5524415; doi:10.1371/journal.pgen.1006892)
Supplement: S1 Table — (DOCX) [file pgen.1006892.s006.docx]

**S1 Table. Metal levels in adult zebrafish tissue**

|  | **Mn**  **(μg/g)** | **Fe**  **(μg/g)** | **Zn**  **(μg/g)** | **Cu**  **(μg/g)** | **Ni**  **(μg/g)** | **Co**  **(μg/g)** | **Mg**  **(μg/g)** | **Cd**  **(μg/g)** | **Pb**  **(μg/g)** |
| --- | --- | --- | --- | --- | --- | --- | --- | --- | --- |
| **WT male** | 5.18 ±1.41 | 75.80 ±33.09 | 231.60±13.39 | 2.94±0.36 | 6.36±1.08 | 0.41±0.05 | 808.86±50.24 | 0.17±0.08 | 0.56±0.02 |
| **WT female** | 4.06±0.74 | 58.42 ±10.54 | 180.20±17.11 | 2.81±0.17 | 3.62±0.39 | 0.27±0.03 | 794.89±38.16 | 0.17±0.02 | 0.57±0.01 |
| **Mut male** | 8.74±0.97 | 77.84 ±14.56 | 282.20±17.73 | 2.62±0.68 | 6.86±0.71 | 0.37±0.05 | 822.94±10.55 | 0.21±0.05 | 0.58±0.03 |
| **Mut female** | 7.62 ±0.86 | 50.68 ±10.09 | 198.40±15.03 | 2.15±0.73 | 4.18±0.31 | 0.26±0.03 | 896.51±50.04 | 0.18±0.25 | 0.56±0.06 |
